# Neddylation-dependent protein degradation is a nexus between synaptic insulin resistance, neuroinflammation and Alzheimer’s disease

**DOI:** 10.1186/s40035-021-00277-8

**Published:** 2022-01-06

**Authors:** Alessandro Dario Confettura, Eleonora Cuboni, Mohamed Rafeet Ammar, Shaobo Jia, Guilherme M. Gomes, PingAn Yuanxiang, Rajeev Raman, Tingting Li, Katarzyna M. Grochowska, Robert Ahrends, Anna Karpova, Alexander Dityatev, Michael R. Kreutz

**Affiliations:** 1 RG Neuroplasticity, Leibniz-Institute for Neurobiology, 39118 Magdeburg, Germany; 2 German Center for Neurodegenerative Diseases (DZNE), 39120 Magdeburg, Germany; 3 Center for Behavioral Brain Sciences, Otto Von Guericke University, 39120 Magdeburg, Germany; 4 Leibniz-Institut Für Analytische Wissenschaften-ISAS-e.V., 44227 Dortmund, Germany; 5 Leibniz Group ‘Dendritic Organelles and Synaptic Function’, Center for Molecular Neurobiology, ZMNH, University Medical Center Hamburg-Eppendorf, 20251 Hamburg, Germany; 6 Department of Analytical Chemistry, Faculty of Chemistry, University of Vienna, 1090 Wien, Austria; 7 Medical Faculty, Otto-von-Guericke University, 39120 Magdeburg, Germany

**Keywords:** Metabolic syndrome, Alzheimer's disease, Neddylation, Cullins, MLN-4924, Insulin, IRS1, Amyloid-β, TNFα

## Abstract

**Background:**

The metabolic syndrome is a consequence of modern lifestyle that causes synaptic insulin resistance and cognitive deficits and that in interaction with a high amyloid load is an important risk factor for Alzheimer's disease. It has been proposed that neuroinflammation might be an intervening variable, but the underlying mechanisms are currently unknown.

**Methods:**

We utilized primary neurons to induce synaptic insulin resistance as well as a mouse model of high-risk aging that includes a high amyloid load, neuroinflammation, and diet-induced obesity to test hypotheses on underlying mechanisms.

**Results:**

We found that neddylation and subsequent activation of cullin-RING ligase complexes induced synaptic insulin resistance through ubiquitylation and degradation of the insulin-receptor substrate IRS1 that organizes synaptic insulin signaling. Accordingly, inhibition of neddylation preserved synaptic insulin signaling and rescued memory deficits in mice with a high amyloid load, which were fed with a 'western diet'.

**Conclusions:**

Collectively, the data suggest that neddylation and degradation of the insulin-receptor substrate is a nodal point that links high amyloid load, neuroinflammation, and synaptic insulin resistance to cognitive decline and impaired synaptic plasticity in high-risk aging.

**Supplementary Information:**

The online version contains supplementary material available at 10.1186/s40035-021-00277-8.

## Background

Modern lifestyle has led to a sharp increase in the elderly population and a growing prevalence of metabolic dysfunction. More than 1.4 billion adults worldwide are overweight, with a prevalence of obesity of 20% in Organisation for Economic Co-operation and Development countries and almost 40% in the USA [1]. The metabolic syndrome (MetS), which is characterized by overweight, insulin resistance, high glucose levels and hypertension, has become a major threat to healthy living and active aging. Of note in this regard, the MetS is also associated with a higher risk of late-onset Alzheimer’s disease (LOAD) [2–6]. Individuals diagnosed with MetS show a greater risk of developing cognitive decline later in life [3, 7–9] and LOAD patients tend to have an even poorer prognosis when MetS is diagnosed as well [3, 10, 11]. Accordingly, early epidemiological studies suggested that a reduction of prevalence of MetS symptoms by 25% would reduce the incidence of LOAD in a population with high amyloid load by 20% [12].

The MetS has been associated with impaired performance in different cognitive domains [8], some of which are associated with the hippocampus-dependent learning and memory [13, 14]. Accordingly, synaptic insulin resistance (IR) in the hippocampus is one of the hallmarks of cognitive decline in MetS [13, 15, 16]. It has been suggested that IR and amyloid-β (Aβ) pathology interact functionally in Alzheimer’s disease (AD) [10, 17–19] and that their interaction might cause worsening of AD symptoms. However, this notion is based on several unknowns. The underpinnings of the molecular interplay between IR and Aβ pathology are, for instance, not well understood, in part because the topology and regulation of synaptic insulin signaling pathways are not well investigated. Along these lines, the mechanisms underlying synaptic IR are not entirely clear yet.

Numerous studies have demonstrated that chronic neuroinflammation is prominently present in the brains of AD patients [20–22] and previous work has proposed a link between elevated levels of proinflammatory cytokines and Aβ-induced synaptic dysfunction [23]. In particular, tumor necrosis factor-α (TNFα) has deleterious effects on the plasticity and integrity of spine synapses [23]. A causal link of Aβ-induced synaptic dysfunction to neuroinflammatory processes is very likely since published data suggest that a 'western diet' promotes inflammatory processes in the brain very similar to those described for Aβ pathology [11, 24, 25]. Collectively, the evidence points to a pathological triad consisting of synaptic IR, chronic neuroinflammation and Aβ deposition as a hallmark for high-risk aging. In the present study, we identified a nodal point of interaction of all three conditions that might be used as a target for intervention. To that end, we established in vitro and in vivo models to examine interactions occurring at the prodromal phase of LOAD, including a transgenic mouse model of high cerebral amyloid levels as a predisposing environment.

## Methods

### Neuronal cell culture

Rat neuronal primary cultures were prepared as described previously [26]. Hippocampal or cortical neurons at 21-days in vitro (DIV) were treated for 24 h with 100 nM insulin, 10 ng/ml TNFα, or a combination of both to induce IR in vitro. Glial release of TNFα induces neuroinflammation [23]. Therefore during the preparation of neuronal primary culture, the addition of Ara-c (cytosine β-*D*-arabinofluranoside), a suppressor of glial proliferation, was omitted. Before application, half of the conditioned Neurobasal Medium (NB, Gibco, Gaithersburg, MD) was replaced with fresh serum-free NB medium. To assess responsiveness to insulin, cell were washed with conditioned NB medium for 1 h and either 100 nM insulin or vehicle was applied for 15 min.

### TBA2.1 mice and MetS

Mice were housed in groups of up to 4 in individually ventilated cages (IVCs, Green line system, Tecniplast, Lugano, Switzerland) under controlled environmental conditions (22 ± 2 °C, 55% ± 10% humidity, 12 h light/dark cycle, with lights on at 06:00). Animals had free access to food and water. Male wild-type (WT, +/+) and heterozygous (+ /Tg) TBA2.1 mice [27] were fed with normal chow until the age of 8–9 weeks. After this period, they were fed with either a regular diet (RD) or a high-fat/high-calorie chow (High-fat diet [HFD], Ssniff #E15126-34, Soest, Germany). Mice fed with HFD were selected for further experiments when they reached a 70% body weight increase, which took on average 4–5 months. Mice used in the experiments were 6–8 months old.

### Aβ3(pE)-42 oligomer and drug treatment

Aβ3(pE)-42 oligomers (Anaspec, #AS‐20,276; Fremont, CA) were prepared according to a previously established protocol [28]. The lyophilized peptide was dissolved in 1,1,1,3,3,3-hexafluoro-2-propanol (HFIP) to 0.5 mg/ml and the aliquots were stored at − 80 °C. HFIP was evaporated for 24 h at room temperature. The peptide was dissolved in 0.1 M NaOH, diluted in NB medium buffered with 0.1 M HCl, and incubated for 24 h. The oligomers were added directly to cultures at a final concentration of 500 nM [24]. The neural precursor cell-expressed developmentally down-regulated gene 8 (NEDD8) inhibitor MLN-4924 (#A-1139, Active Biochem, Hong Kong, China) was directly applied to the culture medium at a final concentration of 1 μM [29]. MG-132 (#M-1157, AG Scientific, San Diego, CA) was used in cell culture experiments at a final concentration of 20 µM.

### Expression constructs and Adeno-associated virus 9 (AAV9) infection

Expression constructs are listed in Additional file 1: Table S1. Cortical neurons cultured in T-75 flasks (#156499, Thermo Scientific, Freiburg im Breisgau, Germany) were infected at DIV10 with 10,000 vg/cell of AAV9 expressing either HA-NEDD8 or the HA tag alone for 14 days. Thereafter, neuronal cell lysates were used for tag-specific immunoprecipitation with anti-HA microbeads (µMACS™ HA Isolation Kit, #130-091-122, Miltenyi Biotec, Bergisch Gladbach, Germany).

### Glucose tolerance test

A small drop of blood (< 5 μl) was taken from the tail vein of the animals and placed on the test strip of the blood glucose meter Contour Next (Bayer, Leverkusen, Germany). Glucose in the blood measured after 6-h fastening was considered as the basal level (t = 0). Immediately after the first measurement, *D*(+)glucose solution of 0.25 g/ml was intraperitoneally injected into the animals at a final concentration of 1 g/kg. Blood glucose levels were measured at 15, 30, 60 and 120 min (t = 15, t = 30, t = 60 and t = 120) postinjection.

### Enzyme-linked immunosorbent assays (ELISA)

Animals were anesthetized with ketamine (100 mg/kg) and xylazine (10 mg/kg). Blood was collected directly from the heart ventricle. Following blood coagulation at room temperature, samples were centrifuged for 10 min at 1500 *g* and the blood serum was collected. Serum insulin levels were measured using a mouse insulin ELISA kit (#EZRMI-13 K; Merck Millipore, Darmstadt, Germany). To determine the TNFα level in brain tissue, frozen cortices from WT and heterozygous TBA2.1 mice fed with either RD or HFD were homogenized in ice-cold PBS supplemented with protease inhibitor cocktail. After two freeze–thaw cycles, the homogenates were centrifuged for 5 min at 5000 *g* at 4 °C. The supernatant was assayed immediately after isolation using a mouse TNFα ELISA kit (#MHSTA50; R&D Systems, Wiesbaden, Germany) according to the manufacturer’s instruction.

### Behavioral experiments

Novel location recognition and novel object recognition experiments were performed in a square arena (50 × 50 × 50 cm^3^) under mild light conditions as described previously [30]. The task consisted of 4 sessions: habituation, training, novel location recognition and novel object recognition. On the first day, the animals were habituated to the empty arena for 20 min. The training session took place 24 h later where the mice were left free to explore for 20 min a pair of similar objects (made of plastic mounting bricks), positioned in the arena. Twenty-four hours later, one of the identical objects was moved to a new position, and mice were left for 20 min in the arena for exploration. After the last 24-h interval, a novel object recognition test was performed, in which a familiar object was replaced by a novel one and free exploration was observed for 20 min. In studies of the effect of MLN-4924 in HFD-fed mice, the exploration time in all sessions was set to 10 min. All four sessions were video-recorded, and behavior was analyzed offline using ANY-maze software (ANY-MazeTM Video Tracking System, version 4.50/4.99, Stoelting Co., Wood Dale, IL). Exploration was considered only when the animal touched or reached the objects with nose at a distance of less than 2 cm. The time mice spent exploring the objects was recorded, and the discrimination index was calculated, taking into account the difference of time spent exploring the new and the familiar object locations ((T_new_ − T_familiar_)/(T_new_ + T_familiar_) × 100%). Chambers and objects were cleaned with 10% ethanol before and after each animal was tested.

### MLN-4924 administration in mice

MLN-4924 was solubilized in DMSO at a concentration of 25 mg/ml. To increase the solubility of MLN-4924 in aqueous buffer, the solubilized drug was mixed with a solution of 10% non-toxic hydrophilic solubilizer 2-hydroxypropyl-β-cyclodestrin (#12446-35-5; Sigma-Aldrich Chemie, Hamburg, Germany) to a final concentration of 0.5 mg/ml and sterile filtered. A dose of 2 mg/kg of MLN-4924 or vehicle was injected intraperitoneally once a day for 14 days.

### Acute hippocampal slice preparation and electrophysiology

Hippocampal slices from male WT (+/+) and heterozygous TBA2.1 (Tg) mice were prepared according to previously described protocols [28, 29]. Field excitatory postsynaptic potentials (fEPSPs) were recorded with glass capillary microelectrodes (3–5 MΩ) filled with artificial cerebrospinal fluid (aCSF), amplified by an Extracellular Amplifier (EXT-02B, NPI electronic, Germany) and digitized at a sampling frequency of 5 kHz by Digidata 1401plus AD/DA converter (CED, England). Stimulation strength was adjusted to 40%–50% (long-term depression, LTD) of the maximum fEPSP-slope values. A single stimulus with 0.1 ms width was applied every 30 s (at 0.0333 Hz) and values were averaged every 3 min. Following 30-min stable baseline recording, a low-frequency stimulus (900 stimuli at 1 Hz frequency) was applied for induction of LTD. For bath application of MLN-4924, the drug was solubilized in DMSO and diluted in aCSF at a final concentration of 1 μM. Following 10-min baseline recording, MLN-4924 or DMSO was applied 20 min before LTD induction and kept for the entire duration of recording.

### Immunoprecipitation of phosphatidylinositide 3-kinase γ (PI3Kγ) and PI3Kγ ELISA

PI3Kγ was immunoprecipitated either from cultured primary cortical neurons or hippocampi of TBA2.1 mice. Cultured cells were collected in 20 mM Tris–HCl, pH 7.4, 137 mM NaCl, 1 mM CaCl_2_ 1 mM MgCl_2_, and 1 mM Na_3_VO_4_, and pelleted at 500 *g*. Dissected mice hippocampi were snap-frozen in liquid nitrogen, mechanically homogenized using a pellet pestle motor (Kimble Kontes, Vineland, NJ) and pelleted at 500 *g*. Both types of samples were lysed for 1 h in lysis buffer (20 mM Tris–HCl, pH 7.4, 137 mM NaCl, 1 mM CaCl_2_ 1 mM MgCl_2_, 1 mM Na_3_VO_4_, 1% NP-40, 1 mM PMSF) and then centrifuged at 14,000 *g* to sediment the insoluble material. The supernatant was incubated with 2 µg of rabbit polyclonal anti-PI3Kγ antibody (#5405; Cell Signaling Technology, Frankfurt, Germany) with gentle rotation for 1 h at 4 °C. Secondary antibody-bound dynabeads protein G (#10003D; Thermo Fisher Scientific, Schwerte, Germany) was incubated with the lysate containing the primary antibody-bound PI3Kγ for 1 h at 4 °C. The beads were collected with the use of DYNAL magnet (Invitrogen, Schwerte, Germany) and subsequently washed with the lysis buffer followed by wash buffer (0.1 M Tris–HCl, pH 7.4, 5 mM LiCl, 1 mM Na_3_VO_4_), TNE buffer (0.1 M Tris–HCl, pH 7.4, 5 mM LiCl, 1 mM Na_3_VO_4_) and an ELISA-reaction buffer (20 mM Tris–HCl, 4 mM MgCl_2_, 10 mM NaCl, ATP 150 μM). While still bound to the beads, PI3Kγ reaction was set up by adding the reaction buffer supplemented with 10 μM of PI(3,4)P_2_ and incubation at 37 °C for 2 h. In the negative control samples, 10 nM of AS-605240 was added to inhibit PI3Kγ activity. The kinase reaction was stopped using the kinase stop solution (reaction buffer supplemented with 4 mM EDTA). Reaction buffer containing the product of the enzymatic reaction phosphatidylinositol (3,4,5)-triphosphate (PI(3,4,5)P_3_) was collected and used for a competitive ELISA (PI3-Kinase Activity ELISA: Pico, #K-1000S, Echelon Biosciences, Göttingen, Germany) according to the manufacturer’s instructions. Thereafter, PI3Kγ was extracted from the beads using 2× SDS buffer and used for immunoblotting.

### Synaptosome preparation

Synaptosomes were prepared from mouse cortices or primary rat cortical culture (21 DIV) according to previously published protocols [31, 32]. The cortex from mice was used in toto. To assess synaptic responsiveness to insulin, isolated synaptosomes from +/+ and +/Tg TBA2.1 mice on RD or HFD were stimulated with 100 nM of insulin or vehicle for 10 min at 37 °C. The reaction was performed in HEPES-buffered Krebs-like buffer (HBK, containing 308 mM NaCl, 308 mM KCl, 154 mM MgSO_4_, 1 M CaCl_2_, 100 mM Na_2_HPO_4_, 87 mM HEPES/Tris, pH 7.4, 0.48 g *D*(+)-glucose) supplied with 8 mM of ATP (#A2383; Sigma-Aldrich Chemie, Hamburg, Germany). Synaptosomes were centrifuged at 10,000 *g*, washed twice with fresh buffer and finally lysed in 2× SDS buffer.

### Immunocytochemistry

Neurons were fixed in 4% paraformaldehyde (PFA) at room temperature. After permeabilization with 0.1% Triton-X/PBS, cells were blocked in blocking buffer (2% glycine, 2% BSA, 0.2% gelatin and 50 mM NH_4_Cl) and subsequently probed with primary and secondary antibodies (Additional file 1: Table S1) as described previously [32]. Coverslips were mounted with Mowiol (# 17951500; Polysciences Inc., Hirschberg an der Bergstraße, Germany).

### Immunohistochemistry

Mice were anaesthetized with isofluran (Baxter Deutschland GmbH, Unterschleißheim, Germany) and then perfused with 0.9% NaCl followed by fixation with 4% PFA in PBS. Brains were processed as described previously [33]. Briefly, slices were incubated in immunohistochemistry blocking buffer (10% goat serum, 0.3% Triton-X 100 in PBS) and subsequently with primary antibody (Additional file 1: Table S1) diluted in blocking buffer. Hippocampi of both hemispheres from mouse brain sections were imaged on a SP5 CLSM system (Leica Mycroystem, Mannheim, Germany). Maximum projections of the scans, comprised of 5 z-stacks (0.39 μm z-step), were created using ImageJ software (NIH, Bethesda, MD) for subsequent analysis. The region of interest (ROI) was defined by positioning a rectangular frame of 220 × 300 μm^2^ onto the pyramidal cell layer of CA1 adjacent to the fasciola cinereum at low-power magnification (20×). Three to four brain sections from each animal were used for quantification and the number of positive cells for each marker (Iba1, GFAP, NeuN) was counted.

### Fluorescence microscopy and image analysis

Image analysis was carried out using a Zeiss Axio Imager A2 fluorescent microscope (Zeiss, Jena, Germany) with Cool Snap EZ camera (Visitron System, Puchheim, Germany) and MetaMorph Imaging software (MDS Analytical Technologies, Ismaning, Germany). Up to 3 coverslips were treated individually and processed per group. For each coverslip, the same exposure time and intensity were taken among the different groups. After background subtraction, the fluorescence intensity of the immunosignal was measured along dendrites right after the first branching point using ImageJ software. The synaptic immunofluorescence intensities of pan-AKT and phospho-AKT were assessed in a region of 400 nm × 400 nm square set by the mask generated based on synaptic marker Shank3. The Shank3 mask was created semi-automatically using OpenView software [34].

### Confocal laser scan microscopy

Images were acquired using Leica SP8 TCS STED 3X confocal microscopy, equipped with a pulsed White Laser (WLL) and a diode 405 nm laser. To quantify changes of pAKT and AKT in the spines, dendrites were scanned sequentially with detection of Alexa fluorophore (AF) 488 for MAP2 or GFP, AF 568 and AF 633 for Shank3. The optical sections were acquired along Z-axis with 0.27-μm Z resolution. After background subtraction, the fluorescent intensity was measured within ROI defined by Shank3 mask using ImageJ software.

### Heterologous co-immunoprecipitation, pull-down assays and immunoblotting

Human embryonic kidney-293-T (HEK293T) cells were transfected using published protocols [32] and heterologous co-immunoprecipitation was performed as described previously [26]. For insulin receptor substrate 1 (IRS1) ubiquitination analysis, cells were preincubated overnight with MLN-4924 (1 μM) or vehicle. They were then harvested in ice-cold PBS supplemented with protease inhibitor cocktail. For pull-down assay, cells were pelleted and lysed in RIPA buffer (50 mM Tris–HCl, pH 8.0, 150 mM NaCl, 1% NP40, 0.5% Na-deoxycholate, 0.1% SDS) containing protease inhibitor cocktail and the de-ubiquitination inhibitor PR-619 (#662141; Sigma-Aldrich, Hamburg, Germany). Following sonication and centrifugation for 10 min at 14,000 *g*, the supernatant was incubated with 20 μl of Chitin resin (#S6651L; New England Biolabs, Frankfurt am Main, Germany) while rotating for 2 h at 4 °C. The lysate was then transferred to BioSpin Chromotographic columns (#732-6204; Bio-Rad, Feldkirchen, Germany) and washed with RIPA buffer (50 mM Tris–HCl, pH 8.0, 500 mM NaCl, 1% NP40, 0.5% Na-deoxycholate, 0.1% SDS). Protein complexes were eluted with 2× SDS buffer. Both 20 μl of input and the eluate were loaded onto SDS-PAGE gradient gels as described previously [32]. Images were acquired using Intas ECL Chemocam Imager (Li-cor, Bad Homburg vor der Höhe, Germany). Protein band intensities were measured using Gel Analyzer plugin ImageJ software (NIH, Bethesda, USA) and normalized to the loading control.

### Statistics

Statistical analysis was carried out with GraphPad Prism software (GraphPad software Inc., San Diego, CA). Data are presented as mean ± SEM. Student’s *t*-test (two experimental groups) or one-way ANOVA followed by Tukey’s multiple comparison test was used for comparison as denoted in figure legends. For biochemical and behavioral analysis, a two-way ANOVA was employed followed by Bonferroni's multiple comparison test. For LTD experiments, averages from 180 to 210 min after LTD induction were compared by two-way ANOVA followed by Bonferroni's multiple comparison test.

## Results

### Induction of synaptic IR

**Fig. 1:**
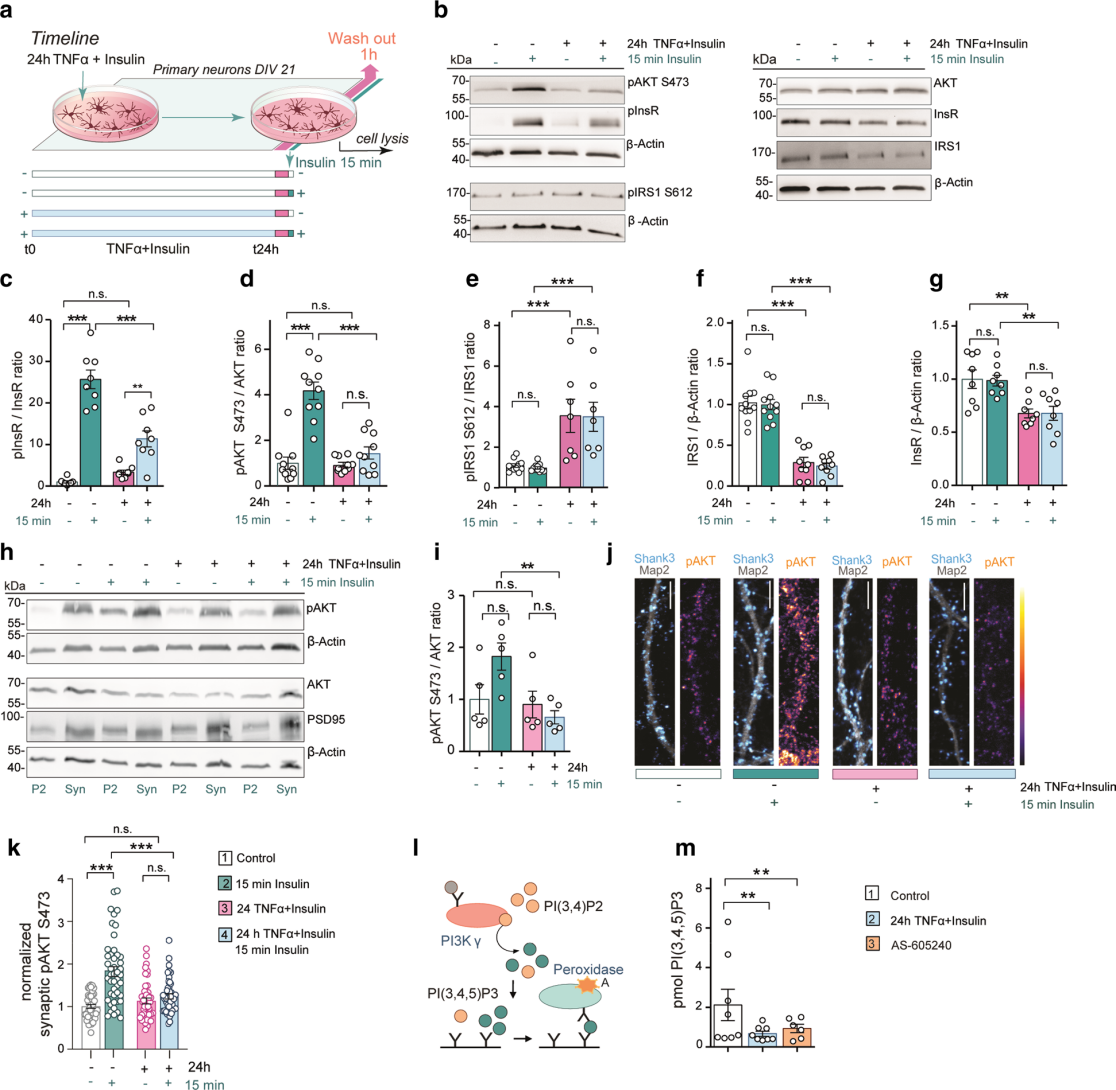
Induction of neuronal insulin resistance (IR) in 21DIV primary neuronal cultures. **a** The schematic illustrates the protocol to induce IR in primary cortical and hippocampal neurons. Representative immunoblots (**b**) probed with antibodies detecting pAKT/pan-AKT, pInsR/pan-InsR, pIRS1/pan-IRS1; and quantitation of pInsR/pan-InsR ratio (**c**), pAKT S473/AKT ratio (**d**) and pIRS1 S612/IRS1 ratio (**e**) as well as total IRS1 (**f**) and InsR (**g**) protein levels in cortical primary neurons upon indicated treatments are shown. β-Actin was used as internal standard for protein loading. **c** Induction of IR results in a significantly reduced InsR phosphorylation in response to 15-min stimulation with insulin. **d** Scatter dot-bar plots showing significant reduction in pAKT level responsiveness of neurons to 15-min stimulation with insulin upon induction of IR (*n* = 10). Treatment of cortical neurons with insulin/TNFα for 24 h resulted in enhanced levels of pIRS1 S612 (**e**, *n* = 11 for groups 1 and 2; *n* = 7 for groups 3 and 4) and reduced levels of total IRS1 (**f**, *n* = 11 for groups 1 and 2; *n* = 9 for groups 3 and 4). **h, i** Immunoblot analysis of synaptosomes (Syn) and the respective crude membrane fraction (P2) after induction of IR (*n* = 5). **i** Induction of IR significantly reduced responsiveness to insulin as measured by pAKT/AKT ratio in synaptosomal fraction. **j** IR reduced levels of pAKT at synapses labelled with Shank3. Confocal images depict dendrites of hippocampal neurons immunostained with antibodies directed against pAKT S473 and Shank3. Original pixel intensities from 0 to 255 are presented as a gradient lookup table. Scale bar, 10 μm. **k** Scatter dot plot depicting synaptic pAKT immunofluorescence intensity quantified within a Shank3 mask normalized to control. *n* = 45 for groups 1 and 4; *n* = 46 for group 2; *n* = 43 for group 3; *n* numbers refer to the number of dendrite segments from different neurons acquired from at least three independent coverslips. **l** Schematic representation of PI3Kγ activity assay where orange circles represent PI(3,4)P_2_ and green circles represent PI(3,4)P_3_. **m** Quantification of the substrate PI(4,5)P_2_ through colorimetric reaction in PI(3,4,5)P_3_-coated detection plate for competitive binding. Cell extracts treated with the PI3Kγ selective inhibitor AS-605240 during the enzymatic reaction were used as a negative control. *n* = 8 for groups 1 and 2; *n* = 6 for group 3. ***P < 0.001, **P < 0.01, *P < 0.05 versus control, by two-way ANOVA followed by Bonferroni’s *post-hoc* test. Two-tailed Student’s *t*-test was used in **m**. n.s. = non-significant. Data are presented as the mean ± SEM

Studies on molecular mechanisms of synaptic IR are facilitated by simplistic models that still have enough complexity to allow for the generation and verification of hypotheses. In the first set of experiments, we therefore established an in vitro model of synaptic IR in neuronal primary cultures, which included 24-h bath application of insulin and TNFα. Following the activation of TNFα receptor 1, stress kinases such as c-Jun N-terminal kinase (JNK) and IκB kinase induce serine phosphorylation of IRS1 [35] and this has been strongly linked to IR and impaired insulin signaling in neurons [36]. In previous work, we found that the post-translationally modified pyroglutamatylated Aβ3(pE)-42 is taken up by astrocytes and potently induces glial release of TNFα and thereby causes synaptic dysfunction [23]. Aβ3(pE)-42 is prominent in AD [37–39] and known to seed highly toxic oligomers that trigger neuroinflammation and TNFα release [40]. Hence, the combination of insulin and TNFα administration should resemble to a certain degree the condition of HFD-induced prolonged exposure to insulin and Aβ-induced neuroinflammation. Cells were therefore incubated with 100 nM insulin and 10 ng/ml TNFα for 24 h followed by acute stimulation with insulin for 15 min to test for insulin responsiveness (Fig. 1a). One of the hallmarks of IR in response to insulin stimulation in many cell types is the diminished phosphorylation of the insulin receptor (pInsR) and the serine/threonine protein kinase-B/AKT (pAKT) [41]. Quantitative immunoblotting revealed reduced pInsR/InsR and pAKT/AKT ratios in response to 15-min insulin stimulation as a result of combined TNFα and insulin pretreatment, indicative of synaptic IR (Fig. 1b–d).

Stimulation of InsR activates insulin-receptor substrates, such as IRS1, by means of phosphorylation at specific tyrosine residues, resulting in activation of the downstream effectors PI3K and AKT. In contrast, phosphorylation of IRS1 at certain serine residues like Ser612 interrupts insulin signaling and renders the scaffold susceptible to degradation [42–46]. We indeed found an increased ratio of Ser612 phosphorylation as a result of overnight TNFα/insulin treatment (Fig. 1b, e) that was caused by reduced IRS1 protein levels (Fig. 1b, f). Similarly, decreased protein levels of the less abundant family member IRS2 were observed (Additional file 1: Fig. S1a, b). Moreover, the total InsR level was also reduced, further indicating IR (Fig. 1b, g). Collectively, the results suggest that neuronal IR might develop as a consequence of an altered phosphorylation as well as protein degradation of IRS1 and IRS2.

We next isolated synaptosomes from cortical primary neurons treated with insulin and TNFα followed by acute insulin stimulation to test for insulin responsiveness (Fig. 1h). Reduced phosphorylation of AKT confirmed synaptic IR (Fig. 1i). In addition, immunocytochemistry of mature hippocampal primary neurons revealed reduced activation of AKT at postsynaptic sites (Fig. 1j, k) and no changes in total AKT fluorescence intensity (Additional file 1: Fig. S1c, d). The conversion of PI(3,4,5)P_3_ from PI(4,5)P_2_ by PI3K is instrumental for insulin signaling since PI(3,4,5)P_3_ triggers phosphorylation of AKT [47–49]. Accordingly, ELISA assays (Fig. 1l, m) revealed a reduction in the activity of the synaptic isoform PI3Kγ [50] following induction of IR, to a degree comparable to the treatment with the PI3Kγ inhibitor AS-605240 (Fig. 1m). Immunoblotting of the eluted PI3Kγ revealed that the observed differences are not due to the amount of immunoprecipitated PI3Kγ, but indeed relies on the activity of the enzyme (Additional file 1: Fig. S1e, f).

### IRS1 and InsR degradation requires neddylation

**Fig. 2:**
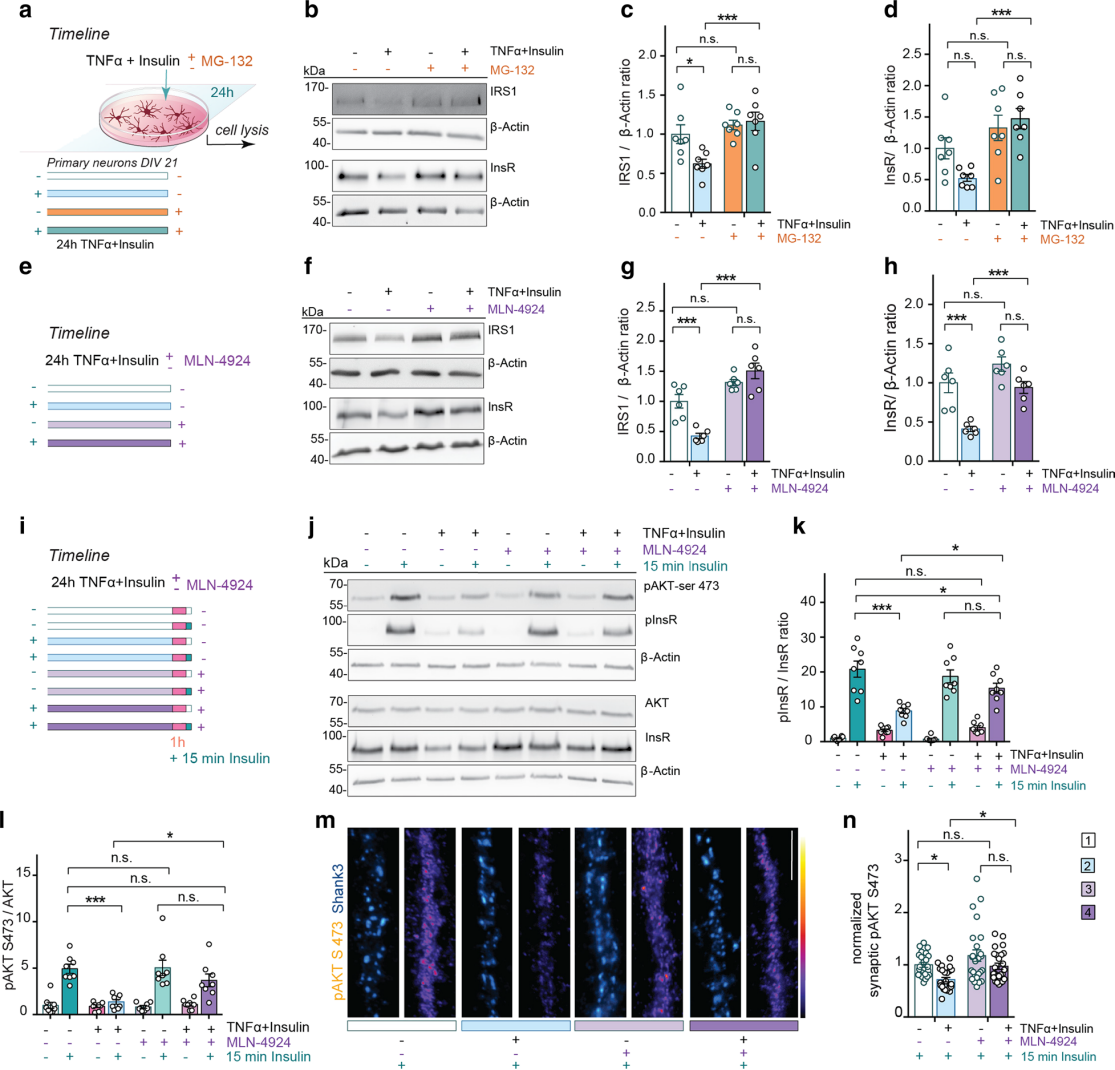
MLN-4924 prevents degradation of IRS1 upon induction of synaptic IR. **a** Schematic drawing represents the timeline of the experiment and corresponding treatments of primary neurons at DIV21. The proteasome inhibitor MG-132 (20 µM) or MLN-4924 (1 µM) was applied for 24 h. **b**, **f** Membranes were probed with anti-IRS1 or anti-InsR antibodies. All blots were probed with β-actin antibody to control for loading. Scatter dot plot represents analysis of IRS1 (*n* = 7, **c**, cortical culture preparations) and InsR (*n* = 7, **d**) levels upon treatment with MG-132, as well as IRS1 (*n* = 6, **g**) and InsR (*n* = 6, **h**) levels upon treatment with MLN-4924. **i** The scheme illustrates the protocol and corresponding treatments. **j** Representative immunoblots of cortical neuron homogenates probed with anti-pAKT S473 and anti-pan-AKT as well as anti-pInsR and anti-pan-InsR antibodies. β-Actin was used as a loading control. pAKT/AKT ratios (*n* = 8, **l**) and pInsR/InsR ratios (*n* = 8, **k**) decreased in response to 15-min stimulation with insulin in conditions of synaptic IR, which was significantly rescued in the presence of MLN-4924. Statistical analysis refers to the groups that have received the acute insulin stimulation only. **m** Representative confocal images of dendrites of primary hippocampal neurons immunolabeled with antibodies against pAKT S473 and synaptic marker Shank3. Original pixel intensities from 0 to 255 are presented as a gradient lookup table. Scale bar, 10 µm. **n** Scatter dot plot representing the mean immunoreactivity of synaptic pAKT. Data were obtained from at least two independent coverslips and at least two independent cell cultures. *n* = 23 for group 1; *n* = 25 for groups 2, 3 and 4. The numbers indicate the number of dendrites acquired from different neurons. ***P < 0.001, **P < 0.01, *P < 0.05 *versus* control, by two-way ANOVA followed by Bonferroni’s *post-hoc* test. n.s. = non-significant. Data are presented as the mean ± SEM

IRS1 degradation could be a nodal point for the induction of synaptic IR. To address whether IRS1 degradation is proteasome-dependent, the proteasome inhibitor MG-132 was co-applied with TNFα and insulin to primary cortical culture (Fig. 2a). Expectedly, IRS1, IRS2 and InsR degradation was counteracted by pharmacological blockade of proteasomal degradation (Fig. 2b–d, Additional file 1: Fig. S2a, b). No regulation of the unrelated Insulin Receptor Substrate p53 (IRSp53) was observed, which served as a negative control in this experiment (Additional file 1: Fig. S2a, c).

Cullins are components of a group of E3-ubiquitin ligases that have been implicated in the degradation of IRS1 [51, 52]. Neddylation, the attachment of the small ubiquitin-like protein NEDD8, is a posttranslational modification of Cullins that is necessary for their activation [53]. Once neddylated, Cullins promote ubiquitination of target proteins for proteasomal degradation. Consequently, we hypothesized that IRS1 might be degraded in a NEDD8-dependent manner. To test this hypothesis, we took advantage of the selective NEDD8-activating enzyme (NAE) inhibitor MLN-4924 [54]. NAE is an E1-like enzyme that catalyzes the first reaction of the neddylation cascade. Similar to MG-132 treatment, bath application of MLN-4924 to primary cortical neurons together with TNFα and insulin for 24 h prevented IRS1, IRS2 and InsR degradation (Fig. 2e–h, Additional file 1: Fig. S2d, e), while it had no effect on levels of IRSp53 (Additional file 1: Fig. S2d, f). We next investigated whether MLN-4924 might also restore the responsiveness to insulin and found that inhibition of neddylation indeed rescued phosphorylation of InsR and AKT (Fig. 2i-l) as well as postsynaptic AKT activation (Fig. 2m, n) in response to 15-min insulin stimulation without affecting the total AKT levels (Additional file 1: Fig. S2g, h).

### Neddylation-dependent IRS1 degradation involves Cullin-7

**Fig. 3:**
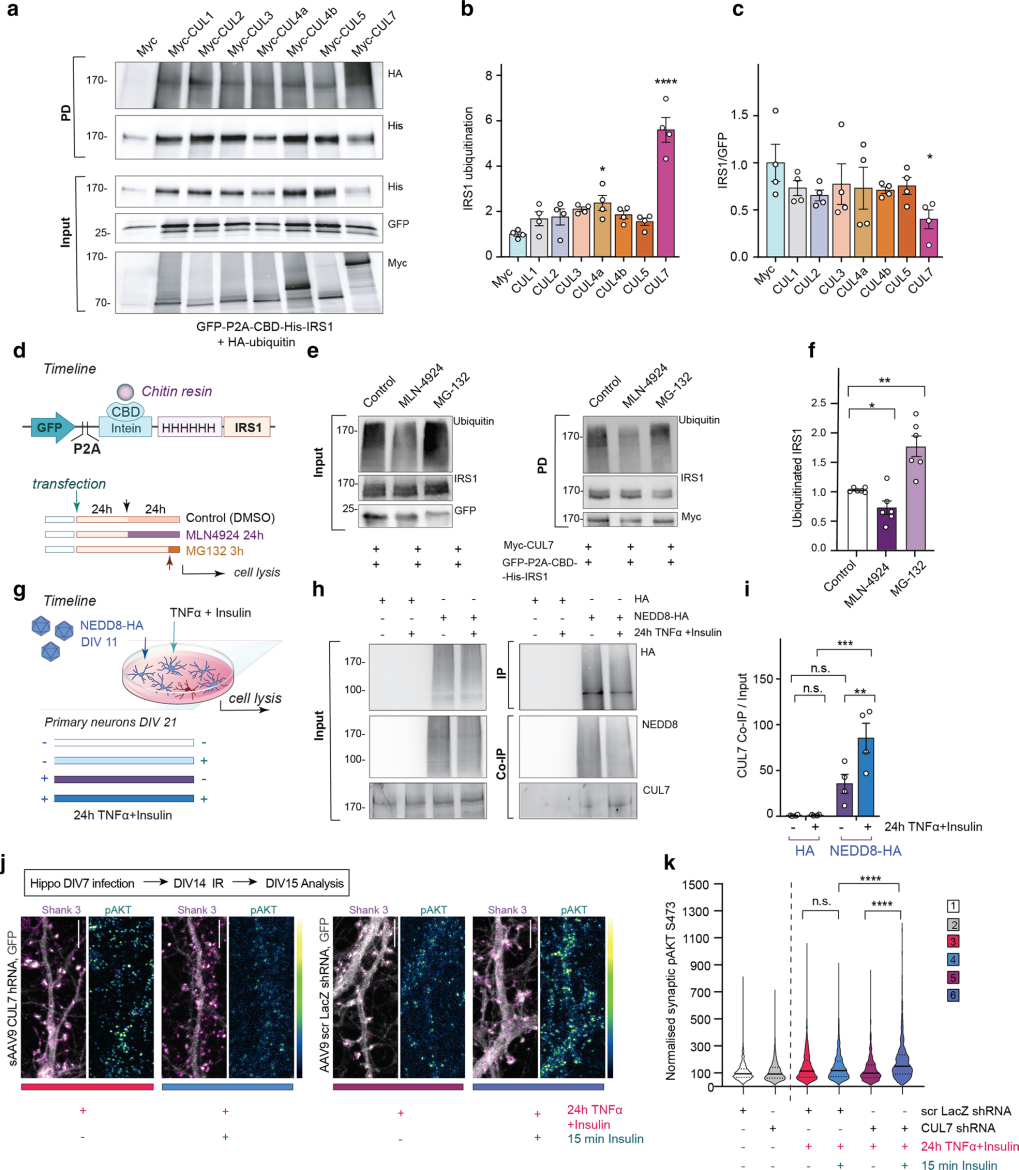
IRS1 ubiquitination and degradation depend on neddylation of CUL7. **a** IRS1 ubiquitination is enhanced in the presence of CUL7. HEK293T cells were co-transfected with GFP-P2A-CBD-His-IRS1, HA-Ubiquitin vectors together with one of the cullin`s family members or empty Myc-vector for control. CBD-His-IRS1 was pulled down with chitin resin for IRS1 enrichment. Quantitation of the levels of pulled-down ubiquitinated IRS1 (**b**, *n* = 4 independent experiments) and of total cell homogenates probed with anti-His antibody, and IRS1 expression was normalized to GFP in **c** (*n* = 4); ****P < 0.0001, *P < 0.05 versus control, by two-way ANOVA followed by Bonferroni’s multiple comparison test. **d** Schematic representation of the expression plasmid and the treatment. **e** Representative immunoblots probed with anti-Ubiquitin, anti-IRS1, anti-Myc, and anti-GFP antibodies. **f** Depicted is a scattered dot plot showing reduced IRS1 ubiquitination in the presence of MLN-4924 (*n* = 6). **g** Schematic of the protocol used to induce IR in DIV11 primary cortical neurons, infected with either AAV9-HA-NEDD8 or AAV9-HA-tag. **h**, **i** Synaptic insulin resistance enhanced the association of NEDD8 with CUL7 as revealed with co-immunoprecipitation experiments from primary neurons infected with HA-NEDD8. **i** Scattered dot plots show quantification of CUL7 in co-IPs normalized to the input (*n* = 4). **j** Representative confocal images of dendrites of primary hippocampal neurons infected with AAV9-shRNA CUL7 and AAV9-scr shRNA and immunolabeled with antibodies against pAKT S473 and the synaptic marker Shank3. Original pixel intensities from 0 to 255 are presented as a gradient lookup table. Scale bar, 5 μm. **k** *n* = 2566 for group 1; *n* = 1171 for group 2; *n* = 1319 for group 3; *n* = 1194 for group 4; *n* = 857 for group 5; *n* = 1059 for group 6. Violin plot representing means of the pAKT at synaptic sites. Numbers refer to the number of spines detected by the Shank 3 mask. ***P < 0.001, **P < 0.01, *P < 0.05 versus control, by two-way ANOVA followed by Bonferroni’s *post-hoc* test. A two-tailed Student’s *t*-test was used in (**f**). n.s. = non-significant. Data are presented as the mean ± SEM

Seven different Cullins are expressed in the brain and it has been suggested that among the various isoforms, Cullin-7 (CUL7) could have a specific role in the degradation of IRS1 at least in non-neuronal cells [51]. To test the hypothesis that CUL7 is a major regulator of neuronal IRS1 ubiquitination, we co-expressed several Myc-tagged Cullins along with a GFP-P2A-CDB-His-IRS1 fusion protein and HA-ubiquitin in HEK293T cells. Subsequently, we pulled down CDB-His-IRS1 with a chitin resin to purify IRS1 and to detect the level of its ubiquitination with anti-HA antibodies (Fig. 3a, b). Strikingly, the level of IRS1 ubiquitination was significantly higher only in the case of co-expression with CUL7 but not of other Cullins (Fig. 3a, b). Concomitantly, the total level of CDB-His-IRS1, as revealed by the ratio of IRS1 and GFP that were expressed from the same plasmid, was significantly lower in the inputs in the presence of CUL7, suggesting IRS1 degradation (Fig. 3c). This indicates that CUL7 might indeed play a major role in the degradation of IRS1.

We next investigated whether IRS1 ubiquitination triggered by CUL7 is regulated by neddylation. To this end, we co-expressed GFP-P2A-CBD-His-IRS1 together with Myc-CUL7 in HEK293T cells, treated them with either MLN-4924 or MG-132 as a positive control and assessed the level of IRS1 ubiquitination following protein pull-down using anti-ubiquitin antibody for detection of endogenous ubiquitin (Fig. 3d–f). Interestingly, inhibition of neddylation reduced IRS1 ubiquitination, indicating neddylation and subsequent activation of CUL7 (Fig. 3e, f). We therefore next infected primary cortical neurons with AAV9 expressing HA-NEDD8 and subsequently performed immunoprecipitation experiments using anti-HA microbeads (Fig. 3g). Interestingly, CUL7 co-immunoprecipitated with NEDD8 in HA-NEDD8 infected neurons (Fig. 3h) and co-immunoprecipitation of CUL7 was significantly enhanced when neurons were treated with TNFα and insulin for 24 h (Fig. 3h, i). In accordance with the role of CUL7 in driving synaptic IR and IRS1 degradation, shRNA knockdown of CUL7 in primary neurons restored synaptic responsiveness to insulin stimulation in conditions of synaptic IR by increasing synaptic pAKT levels (Fig. 3j, k, Additional file 1: Fig. S3a, b).

### Induction of synaptic IR by insulin and TNFα is related to Aβ pathology

We next investigated the individual contribution of elevated insulin or TNFα levels to synaptic IR (Additional file 1: Fig. S3c, d). Quantitative immunocytochemistry revealed that bath application of TNFα or insulin individually already reduced AKT activation, while co-application of both resulted in a further reduction of AKT phosphorylation (Additional file 1: Fig. S3c, d). Interestingly, co-application of Aβ3(pE)-42 and insulin to mixed neuronal cultures containing astroglia resulted in synaptic IR to a similar extent as the combined treatment with TNFα and insulin (Additional file 1: Fig. S3e, f). Moreover, the effects of Aβ3(pE)-42 on pAKT and pIRS1 levels were blocked by bath application of a monclonal neutralizing anti-TNFα antibody (Additional file 1: Fig. S3g–j), indicating that the effects of Aβ3(pE)-42 on synaptic IR are indeed largely mediated by the proinflammatory cytokine TNFα.

### Induction of MetS in TBA2.1 mice can serve as an in vivo model for synaptic IR and amyloidosis in high-risk aging

We therefore next wondered whether neuroinflammation and Aβ pathology in AD interact with MetS to induce synaptic IR in vivo. TBA2.1 mice express Aβ3(pE)-42 under a Thy-1 promoter and homozygous animals show profound AD pathology [27] and very prominent neuroinflammation resembling in this respect human pathology [20]. However, heterozygous mice within the first 6 months of life exhibit no such phenotype, which is probably due to a lower amyloid load [27]. Hence, they might be well suited as a model of Aβ-induced neuroinflammation that is sub-threshold for AD but that might be exacerbated in MetS and accompanied by synaptic IR if mice are fed with a western HFD. Therefore, we were feeding male heterozygous TBA2.1 mice and corresponding WT control mice until they reached a 70% gain in body weight (Additional file 1: Fig. S4a). This regimen resulted in clear indications of MetS in mice of both genotypes fed with a HFD as evidenced by a blunted glucose response to insulin as well as elevated serum glucose, insulin and cholesterol levels (Additional file 1: Fig. S4b–e).

### HFD causes glial activation and induces neuronal cell loss in the CA1 region of hippocampus in TBA2.1 mice

Glial activation is a vital part of the neuroinflammatory response in AD [20, 21] and neuronal cell loss in the hippocampal CA1 region is a hallmark of the early stage of AD [55]. We found a significantly increased number of Iba1-positive microglia and increased GFAP immunoreactivity in heterozygous TBA2.1 mice fed with a HFD as compared to controls (Additional file 1: Fig. S4f–h). In addition, we observed a significant reduction of NeuN-positive neurons in the pyramidal layer of the hippocampal CA1 region (Additional file 1: Fig. S4f, i). Moreover, the levels of TNFα were elevated in TBA2.1 mice irrespective of the diet (Additional file 1: Fig. S4j).

### HFD induces synaptic IR in mice

**Fig. 4:**
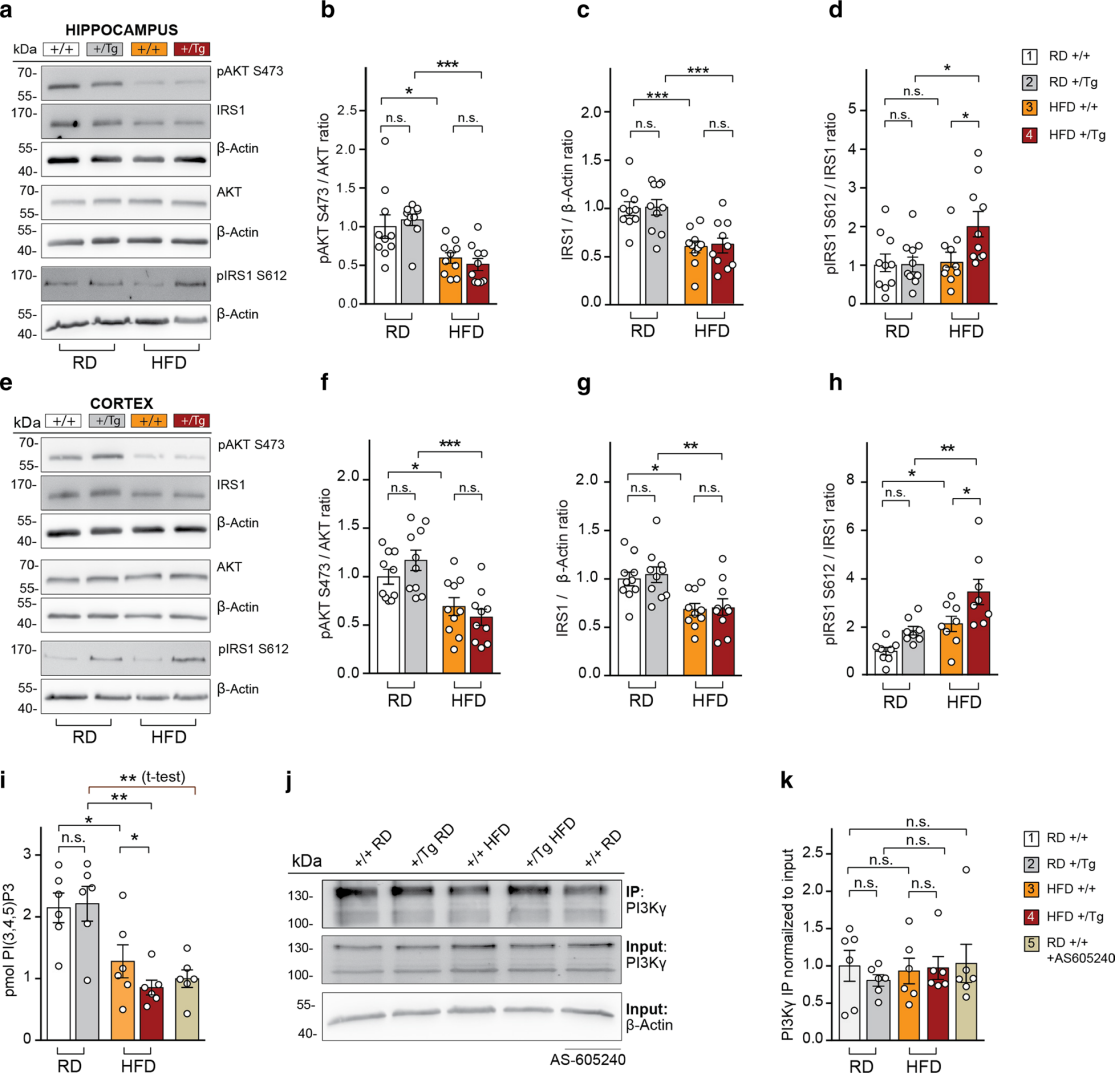
HFD promotes synaptic IR in wild-type and heterozygous TBA2.1 mice. Representative immunoblots of total protein extracts from hippocampus (**a**) and cortex (**e**) of wild-type (+/+) or heterozygous TBA2.1 (Tg) mice fed with a RD or HFD. Membranes were probed with antibodies detecting pAKT (Ser473), AKT, pIRS1 (Ser612) and IRS1 as well as β-actin as a loading control. Scatter dot plots of the normalized pAKT (**b**, **f**), IRS1 (**c**, **g**) and pIRS (**d**, **h**) in hippocampus (*n* = 10) and cortex (*n* = 10). **i** Endogenous PI3Kγ was immunoprecipitated from hippocampal lysates of wild-type and heterozygous TBA2.1 mice fed with a RD or HFD. The amount of PI(3,4,5)P_3_ produced by the enzyme coupled to the beads was determined by ELISA. The inhibitor AS-605240 was added to the wild-type/RD control group and used as a negative control (*n* = 6). **j**, **k** Immunoblot analysis showing no differences in the amounts of immunoprecipitated PI3Kγ between wild-type or heterozygous TBA2.1 mice fed with a RD or HFD (*n* = 6). ***P < 0.001, **P < 0.01, *P < 0.05 versus control by two-way ANOVA followed by Bonferroni’s *post-hoc* test. n.s. = non-significant. Two-tailed Student’s *t*-test was done to compare +/+ RD versus +/+ RD treated with AS-605240 in **i** and **k**. Data are presented as the mean ± SEM

We asked next whether the combination of high amyloid load and HFD can induce synaptic IR. Hippocampi and cortices from WT and heterozygous TBA2.1 mice kept on RD or HFD were dissected and levels of pAKT and pIRS1 as well as the total protein levels of IRS1 were examined (Fig. 4a–h). We observed a substantial reduction of the basal AKT phosphorylation in the hippocampus (Fig. 4a, b) and cortex (Fig. 4e, f) of TBA2.1 mice fed with a HFD. This was accompanied by reduced IRS1 and IRS2 protein levels (Fig. 4a, c, e, g, Additional file 1: Fig S4k–n) and increased phosphorylation of IRS1 at the inhibitory serine residue only in TBA2.1 mice fed with a HFD (Fig. 4a, d, e, h).

In order to address the effects of HFD on synaptic IR, synaptosomes were prepared and insulin responsiveness was tested ex vivo upon stimulation with 100 nM insulin for 15 min in the presence of ATP. As expected, we observed efficient InsR phosphorylation in response to insulin, which was significantly reduced in synaptosomes of mice fed with a HFD (Additional file 1: Fig. S4o–r).

We next performed endogenous immunoprecipitation of PI3Kγ from the total homogenate of heterozygous TBA2.1 mouse hippocampi and PI(3,4,5)P_3_ produced by the enzyme was measured by competitive colorimetric ELISA. The reduced amount of PI(3,4,5)P_3_ converted from the substrate PI(4,5)P_2_ correlates to the reduced enzymatic activity of PI3Kγ in animals who received a HFD (Fig. 4i–k). Interestingly, PI3Kγ activity was even further reduced in TBA2.1 mice fed with a HFD (Fig. 4i–k).

### TBA2.1 mice fed with HFD display  impairments in synaptic plasticity and novel object location/recognition memory

**Fig. 5:**
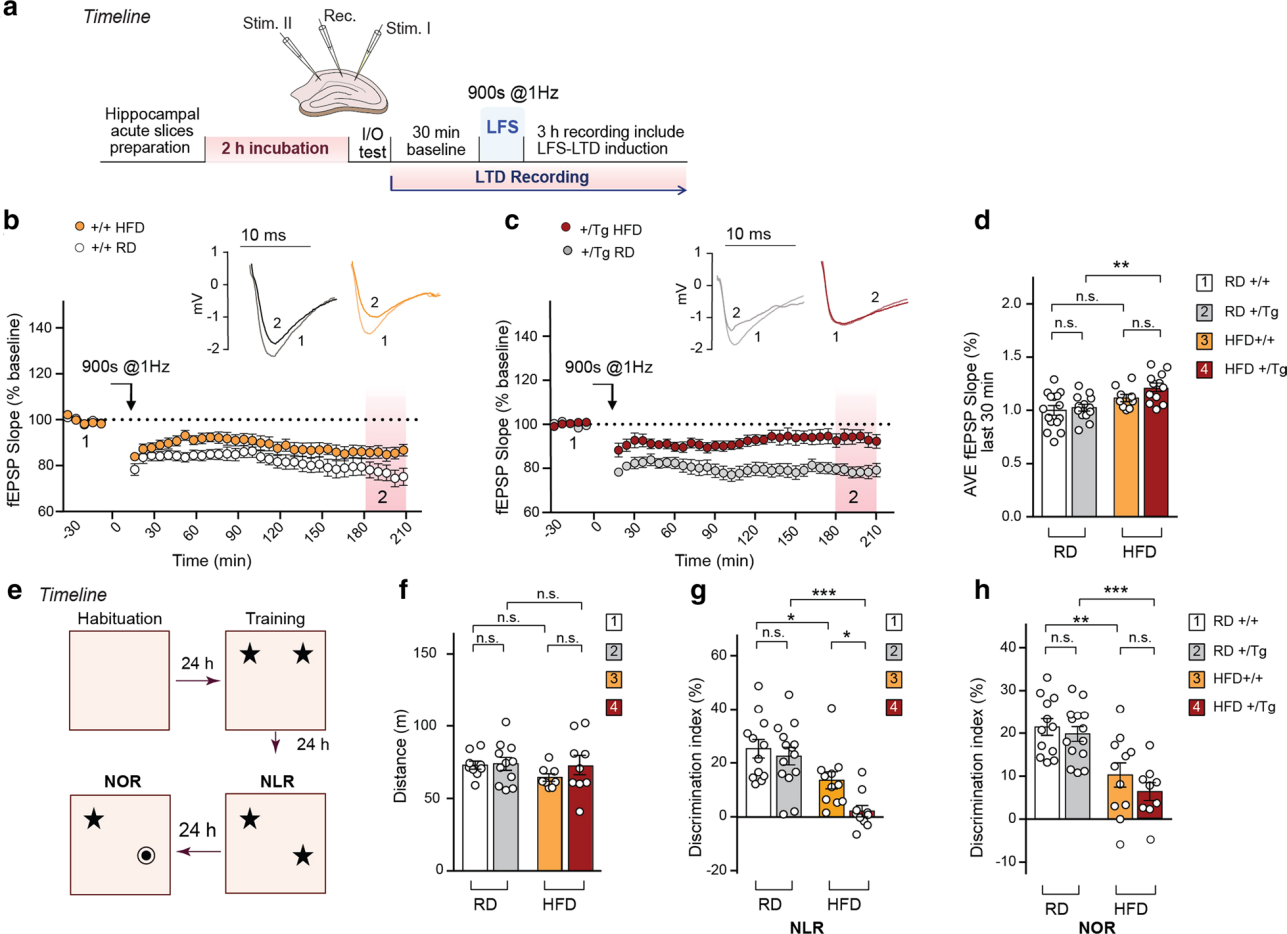
HFD causes impairments in  LTD and novel object location/recognition memory. **a** Schematic illustration of the timeline and LTD induction protocol by low-frequency stimulation (LFS) in acute CA1 hippocampal slices. **b**, **d** Field EPSP recordings in CA1 in acute slices from wt fed with a RD or HFD show no differences between the groups. **c**, **d** LTD induced in slices from heterozygous TBA2.1 mice fed with HFD, shows significant impairment when compared to wild-type (+/+) mice and TBA2.1 (+ /Tg) mice fed with RD. **d** Averaged fEPSP slope values measured 180–210 min after LTD induction. Data were obtained from slices prepared from at least three different mice. *n* = 15 for group 1; *n* = 13 for group 2; *n* = 9 for group 3; *n* = 12 for group 4. **e** Schematic representation of the object location and recognition memory experimental protocol. Scatter dot plot shows the total distance that wild-type and heterozygous TBA2.1 animals fed with a RD or HFD walked during the habituation phase (**f**), the discrimination index of the new located (NLR, **g**) or replaced object (NOR, **h**) as compared to the old one. In **f** *n* = 9 animals in groups 1 and 4; *n* = 11 in group 2; *n* = 8 in group 3. In **g** *n* = 12 for group 1; *n* = 14 for group 2; *n* = 11 for group 3; *n* = 10 for group 4. In **h** *n* = 12 for group 1; *n* = 14 for group 2; *n* = 11 for group 3; *n* = 9 for group 4. ***P < 0.001, **P < 0.01, *P < 0.05 versus control by two-way ANOVA followed by Bonferroni’s *post-hoc* test, n.s. = non-significant

Insulin signaling and PI3Kγ activation in the brain have been shown to be instrumental for the induction of *N*-Methyl-*D*-aspartate receptor (NMDAR)-dependent long-term depression (LTD) in the CA1 region of hippocampus [50, 56]. Therefore, we chose this type of synaptic plasticity to determine the cellular consequences of synaptic IR. To this end, NMDAR-dependent LTD was induced with low-frequency stimulation at Schaffer-collaterals in the CA1 region of acute hippocampal slices (Fig. 5a, Additional file 1: Fig. S5a, b). We found that the fEPSP slope returned to the baseline level following LTD induction only in slices obtained from heterozygous TBA2.1 mice under a HFD regime (Fig. 5b–d).

Learning and memory formation for the spatial localization and recognition of objects requires synaptic plasticity of CA1 neurons and the induction of NMDAR-dependent LTD [57, 58]. When we next tested for such memory tasks in an open field (Fig. 5e), we observed no difference in locomotor activity despite the striking differences in body weight among the groups (Fig. 5f). However, animals subjected to a HFD exhibited a lower discrimination of the newly located (Fig. 5g) and novel object (Fig. 5h) when compared to WT mice fed with a RD. Notably, the discrimination of the newly located object was significantly worse in TBA2.1 mice on HFD as compared to the corresponding control groups (Fig. 5g), which correlates with the impairment in LTD (Fig. 5c, d).

### MLN-4924 injection in TBA2.1 mice ameliorates synaptic IR and prevents HFD-induced synaptic plasticity and memory impairment

**Fig. 6:**
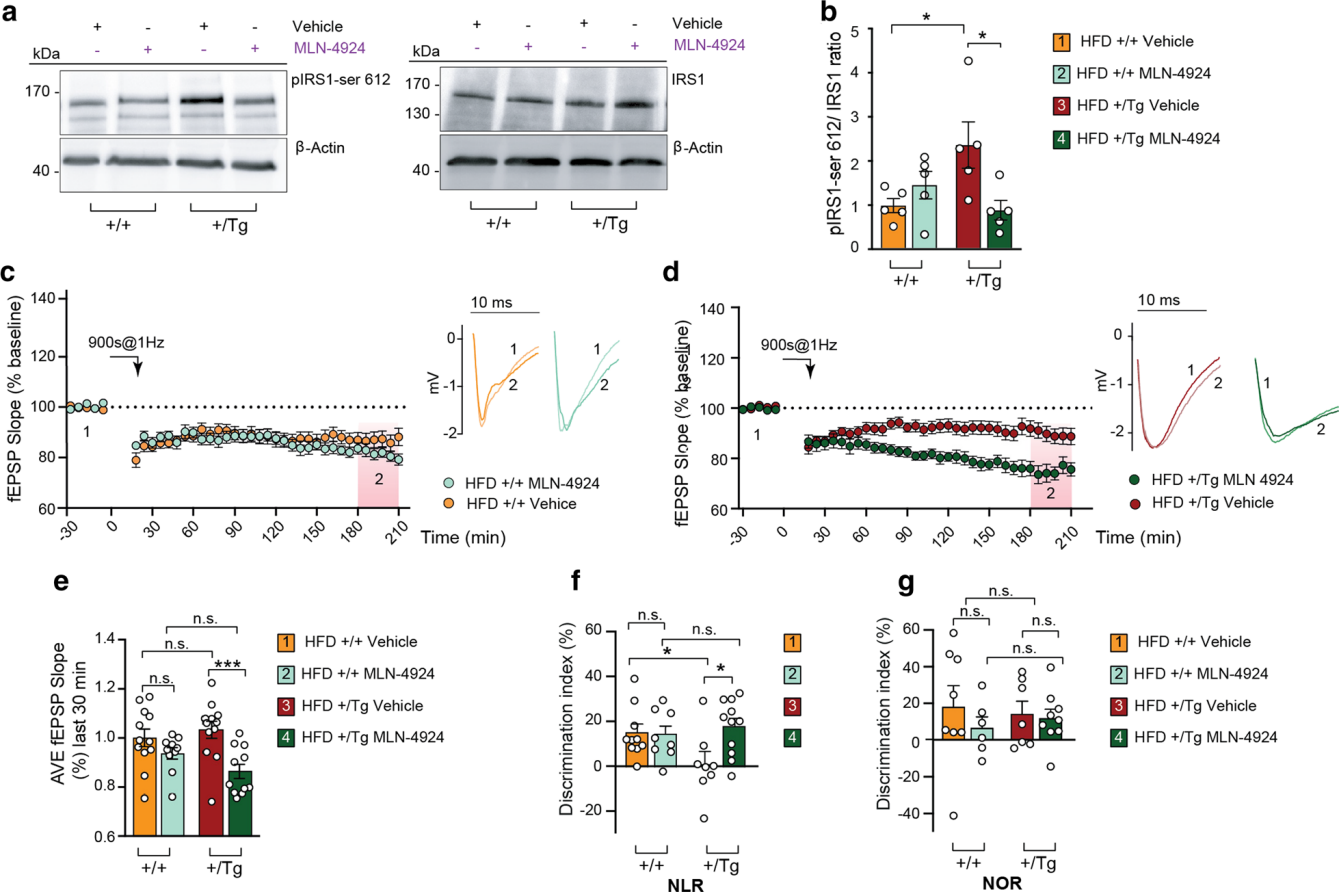
The treatment with MLN-4924 of mice fed with the HFD ameliorates synaptic IR and memory impairment. **a**, **b** Representative immunoblots and quantification of total hippocampal extracts showing a reduction in pIRS1/IRS1 ratio upon the MLN-4924 treatment of wild-type or heterozygous TBA2.1 fed with HFD. IRS1 protein level was normalized to β-actin. *n* = 5. *P < 0.05 versus control, by two-way ANOVA followed by Tukey´s *post-hoc* test. **c**, **d** Quantification of fEPSP slope values in wild-type and heterozygous TBA2.1 mice fed with HFD and injected with MLN-4924 or vehicle shows the rescue of LTD upon the treatment with MLN-4924. **e** Averaged fEPSP slope values measured 180–210 min after LTD induction. *n* = 12 for groups 1 and 3; *n* = 10 for group 2; *n* = 11 for group 4. **f**, **g** Behavioral assessment in mice injected with MLN-4924 or vehicle. **f** Scatter dot plot shows a rescue of the discrimination index of the new located (NLR) without alterations in replaced object location (NOR, **g**). In **f** *n* = 10 for group; *n* = 8 for groups 2 and 3; *n* = 11 in group 4. In **g** *n* = 8 for group 1; *n* = 6 for group 2; *n* = 7 for group 3; *n* = 9 for group 4. *n* indicates the number of animals. ***P < 0.001, *P < 0.05 versus control, by two-way ANOVA followed by Bonferroni’s *post-hoc* test. n.s. = non-significant. Data are presented as the mean ± SEM

In the final set of experiments, we investigated whether the expression of synaptic IR in mice fed with a HFD might be a consequence of neddylation-dependent degradation of IRS1. To study this question, we administered MLN-4924 and analyzed whether this treatment counteracts neuronal IRS1 degradation and protects from cognitive and synaptic plasticity decline. No significant weight loss was observed following MLN-4924 injection (Additional file 1: Fig. S6a) and no indication for alleviating peripheral parameters of MetS was found (Additional file 1: Fig. S6b–e). However, the pIRS1/IRS1 ratio in animals injected with MLN-4924 was reduced, suggesting that the treatment might rescue synaptic IR (Fig. 6a, b). To investigate the effects of MLN-4924 on synaptic function, NMDAR-dependent LTD was induced in the hippocampal CA1 region with low-frequency stimulation of Schaffer-collaterals in acute slices obtained from heterozygous TBA2.1 mice fed with a HFD. In these experiments, we observed a rescue of LTD in obese TBA2.1 mice injected with MLN-4924 as compared to those injected with vehicle (Fig. 6c–e, Additional file 1: Fig. S6f, g). Interestingly, this effect was absent in TBA2.1 mice fed with a regular diet (Additional file 1: Fig. S6h–l). Most importantly, inhibition of neddylation with MLN-4924 improved hippocampal CA1 LTD-dependent learning and memory as evidenced by an enhanced discrimination index in the novel location (Fig. 6f) but not in the novel object recognition task (Fig. 6g). Administration of MLN-4924 did not affect the locomotor activity (Additional file 1: Fig. S6m).

## Discussion

Several studies have shown that synaptic IR in conjunction with chronic inflammation and a high amyloid load is part of a pathological triad that puts individuals at a high risk of developing LOAD with increasing age [10, 17–19]. Impaired neuronal insulin signaling by itself negatively affects cognition and while the underlying mechanisms of synaptic IR are less clear, a number of mechanisms have been proposed for the interaction between neuroinflammation and Aβ-induced synaptic dysfunction [17, 20, 36].

**Fig. 7:**
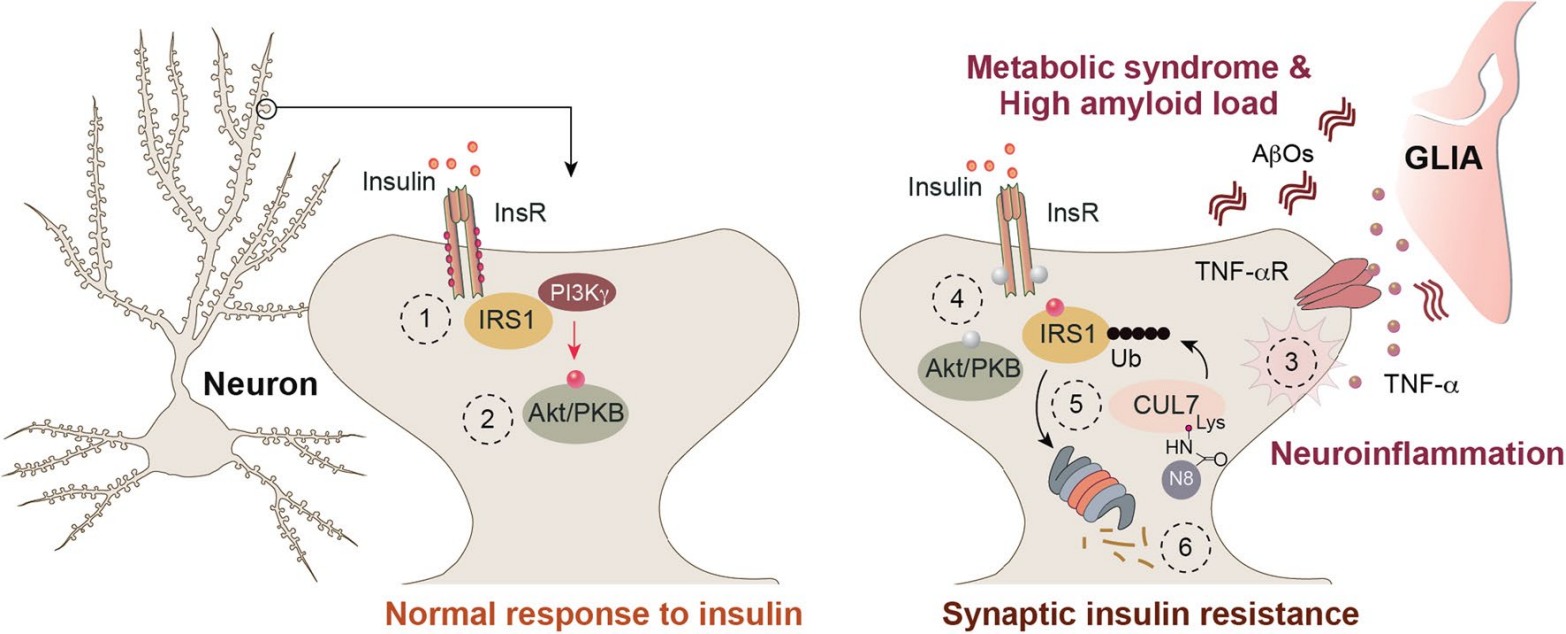
Neddylation-dependent IRS1 degradation is a nodal point in synaptic insulin resistance. Exposure to insulin results in phosphorylation of insulin receptors (InsR) (1) with subsequent activation of ATK/PKB (2). Both high amyloid load *via* stimulation of TNFα release from glia (3)  and MetS alter neuronal responsiveness to insulin (induce synaptic insulin resistance) as evidenced by reduced phosphorylation of InsR and the downstream effector AKT (4) and enhanced phosphorylation of IRS1. In parallel, synergism between MetS, high amyloid load and neuroinflammation promotes neddylation of CUL7 (5) and subsequent degradation of IRS1 (6)

In this study, we report that MetS, neuroinflammation and a high amyloid load essentially converge on neddylation of Cullins and subsequent ubiquitination of the IRS1 scaffold in the hippocampus to induce synaptic IR (Fig. 7), and impairment in LTD and learning of novel object location. Although not directly addressed in this study, we consider it likely that this mechanism might also impact the progression of LOAD. In addition, we found that inhibition of neddylation might be a useful molecular entry point to interrupt this pathological triad. Collectively, the results raise a number of interesting points that we want to discuss in more detail.

### Neddylation and synaptic function

Relatively few studies have addressed the role of neddylation in synaptic function and neddylation in neurons as such has not been studied in much detail yet. Previous work has shown that the synaptic scaffolding protein postsynaptic density-95 (PSD95) is a substrate for neddylation [59] and that neddylation of PSD95 is important for spine stability [59]. In the same study, it was also shown that forebrain-specific knockout of NEDD8-activating enzyme (NAE1) results in several impairments in spine maturation, stability and function [59]. It is therefore no surprise that inhibition of neddylation results in deficits of hippocampal synaptic plasticity [60]. In addition, it was found that synaptic activity regulates neddylation of Cullins in the nucleus and, here in particular Cullin-4B (CUL-4B), which is instructive for the subsequent degradation of the de novo DNA-methytransferase DNMT3A1 [61]. While some reports indicate a synaptic function of Cullins in *Drosophila* [6, 62], the literature on Cullins in mammalian synapses is sparse. We show here that CUL7 seems to play an important role in the induction of synaptic IR (Fig. 7). This is in line with a previous report that showed the involvement of CUL7 in IRS1 ubiquitination in non-neuronal cells [51, 52]. Several studies have proposed mechanisms for the interplay of IR and amyloidosis in AD and its relevance for disease progression [19] and our finding might contribute a novel approach to restoring cerebral insulin function that could offer therapeutic benefits given that neddylation proved to be a druggable target [63]. In addition, our results suggest that peripheral administration of the NAE-1 inhibitor MLN-4924 is in principle feasible to target synaptic processes.

### A novel molecular entry point to treat synaptic IR?

Unfortunately, still very little is known about which synaptic proteins are neddylated and how neddylation affects protein function. An intriguing question is therefore whether and if yes, how neddylation might be regulated at synaptic sites. In a previous study, we observed that activation of glutamate receptor subunit 2A-containing NMDAR at synaptic sites enhances neddylation of CUL4B in the nucleus *via* activation of CaMKIV [61]. Whether the NMDAR Ca^2+^-signal at synaptic sites can also enhance local neddylation of other substrates is currently unknown. In fact, it is even unclear whether the machinery for neddylation is at all present at synapses. NEDD8 has relatively low abundance at synaptic sites as compared to the nucleus [61]. Accordingly, it might be difficult to identify signaling pathways that regulate either directly or indirectly neddylation. In non-neuronal cells, neddylated proteins are prominently associated with the microtubule cytoskeleton [64] and it is likely that apart from Cullins, microtubules and their associated proteins might be a major part of the neuronal neddylome. Administration of the NEDD8-inhibitor MLN-4924 at much higher concentrations than those employed in the present study as well as NAE1 gene knockout results in synaptic loss and impaired synaptic plasticity [59, 65]. It is therefore advisable to identify specific neddylated targets in future work to prevent negative effects of any intervention on synaptic structure and function. As outlined above, one of those could be CUL7. It is involved in IRS1 ubiquitination (Fig. 3a–f) [63] and shRNA knockdown rescued at least partially synaptic IR and we did not observe spine loss or reduced dendrite complexity following protein knockdown (data not shown).

### Cullin7: a nodal point in synaptic IR, neuroinflammation and Aβ-oligomer signaling?

Several signaling pathways have been identified that might contribute to the intricate balance between IRS1 phosphorylation at serine or tyrosine residues, which in turn determines the extent and magnitude of insulin actions [42]. In AD, multiple factors are known to activate the JNK pathway, including Aβ accumulation and TNFα signaling [20, 66]. JNK activation results in IRS1 serine phosphorylation, blocking downstream insulin signaling, and increases the susceptibility of IRS1 to degradation [67, 68]. Impaired insulin signaling in turn accounts for the subsequent activation of glycogen synthase kinase 3 beta and accumulation of Aβ and hyperphosphorylated Tau [68, 69]. In this scenario, the most plausible initial trigger for convergence of pathological events is the facilitation of synaptic IR by TNFα activation of the JNK pathway with the ensued IRS1 inhibition and degradation, which then has a major negative impact on synaptic function, synaptic plasticity, and synaptic connectivity. What is currently missing is a molecular regulatory link between increased neddylation of Cullins and IRS1 degradation under such conditions. An intriguing hypothesis concerns the activation of NAE1 itself by the TNFα/JNK pathway or signaling components of the InsR. Another interesting candidate molecule in this respect is dsRNA-dependent protein kinase (PKR). PKR is activated by Aβ and elevated levels of phophorylated PKR are present in postmortem AD brains [70]. PKR activation occurs in a TNFα-dependent manner and this, in turn, results in IRS1 inhibition, synaptic loss and memory impairment [71]. Most importantly, PKR-knockout mice are resistant to the detrimental effects of Aβ and TNFα [72] and bolstering insulin signaling can counteract the PKR-related AD phenotypes [71]. Thus, PKR might be a key modulator of insulin sensitivity that could underlie memory impairment in MetS and obesity as well as AD and it is likely that a molecular link will exist with other signaling components of the pathological triad consisting of synaptic IR, neuroinflammation and oligomeric Aβ.

## Conclusions

In summary, the present work suggests that neddylation of proteins and in particular Cullins at synaptic sites plays an important role in synaptic IR (Fig. 7). Pharmacological inhibition of neddylation rescues memory impairment and synaptic plasticity in an animal model of high-risk aging and the study paves the way for a systematic investigation of further molecular targets and a deeper appreciation of the interplay between key determinants of lifestyle and age-related pathology.

## Supplementary Information

**Additional file 1. Fig. S1.** Induction of synaptic IR in primary neurons. **Fig. S2.** Proteasomal degradation of IRS2 is not affected by neddylation. **Fig. S3.** CUL7 KD does not alter insulin signaling and Aβ3(pE) application exacerbates IR. **Fig. S4.** MetS induces neuronal cell loss, astroglial and microglial activation in the hippocampal CA1 region of heterozygous TBA2.1 mice. **Fig. S5.** Baseline recordings of fEPSP in hippocampal acute slices from WT and heterozygous TBA2.1 mice fed with a RD or HFD. **Fig. S6.** Characterization of the effects of MLN-4924 on basic metabolic parameters, basal fEPSP slope and LTD-dependent learning and memory in WT and heterozygous TBA2.1 mice fed with a RD or HFD. **Table S1.** Expression constructs and antibodies used in this study.

## Data Availability

All data generated or analyzed during this study are included in this published article and its Additional file 1.

## Abbreviations

AAV9: Adeno-associated virus-9
aCSF: Artificial cerebrospinal fluid
AD: Alzheimer’s disease
Aβ: Amyloid-β
Co-IP: Co-immunoprecipitation
DIV: Day(s) in vitro
ELISA: Enzyme-linked immunosorbent assay
fEPSPs: Field excitatory postsynaptic potentials
GFAP: Glial fibrillary acidic protein
HA-tag: Human influensa hemagglutinin
HFD: High-fat diet
InsR: Insulin receptor
IRS: Insulin receptor substrate
IR: Insulin resistance
JNK: C-Jun-N- terminal kinase
LOAD: Late-onset Alzheimer’s disease
LTD: Long-term depression
MetS: Metabolic syndrome
NMDAR: *N*-Methyl-*D*-aspartate receptor
NAE1: NEDD8-activating enzyme
NEDD8: Neural precursor cell-expressed developmentally down-regulated gene 8
NeuN: Neuronal nuclear antigen and neuron differentiation marker
PI(3,4)P_2_: Phosphatidylinositol (3,4)-biphosphate
PI(3,4,5)P_3_: Phosphatidylinositol (3,4,5)-triphosphate
PKR: Protein kinase R
PSD95: Postsynaptic dendisty-95
TNFα: Tumor necrosis factor α
