# Supplementary material for: Neddylation-dependent protein degradation is a nexus between synaptic insulin resistance, neuroinflammation and Alzheimer’s disease
Source: Transl Neurodegener. 2022 Jan 6;11:2. doi: 10.1186/s40035-021-00277-8 (PMC8734066; doi:10.1186/s40035-021-00277-8)
Supplement: Supplementary file 1 — Additional file 1. Fig. S1. Induction of synaptic IR in primary neurons. Fig. S2. Proteasomal degradation of IRS2 is not affected by neddylation. Fig. S3. CUL7 KD does not alter insulin signaling and Aβ3(pE) application exacerbates IR. Fig. S4. MetS induces neuronal cell loss, astroglial and microglial activation in the hippocampal CA1 region of heterozygous TBA2.1 mice. Fig. S5. Baseline recordings of fEPSP in hippocampal acute slices from WT and heterozygous TBA2.1 mice fed with a RD or HFD. Fig. S6. Characterization of the effects of MLN-4924 on basic metabolic parameters, basal fEPSP slope and LTD-dependent learning and memory in WT and heterozygous TBA2.1 mice fed with a RD or HFD. Table S1. Expression constructs and antibodies used in this study. [file 40035_2021_277_MOESM1_ESM.pdf]

Supplemental Figures and Table

Neddylation-dependent protein degradation is a nexus between synaptic insulin resistance, neuroinflammation and Alzheimer's disease

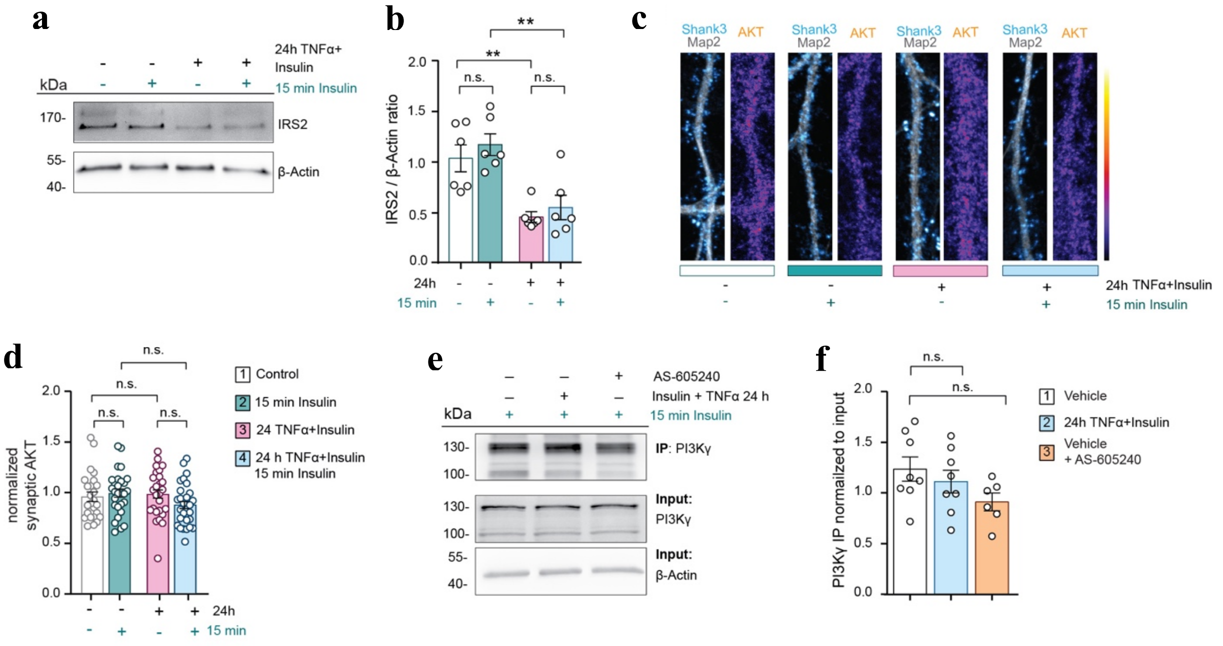

Figure S1

Figure S1. Induction of synaptic IR in primary neurons

a). Immunoblots probed with IRS2 antibody. **b**). Quantitation of IRS2 levels normalized to β-Actin. **c**). Representative images of primary hippocampal neurons immunolabeled with pan-AKT antibodies. Excitatory synapses were identified by Shank3 staining. Original pixel intensities from 0 to 255 are represented as a gradient lookup table. Scale bar is 10 μm. **d**). Scatter dot plot representing no differences in pan AKT immunofluorescence in the Shank3 mask. N-numbers (dendritic segments analyzed) from left to right. *n*=25 for condition 1; *n*= 28 for conditions 2 & 4; *n*=26 for condition 3. **e, f**). Representative immunoblots and analysis of immunoprecipitated PI3Kγ normalized to the respective PI3Kγ input. *n*=8 for condition 1 & 2; *n*=6 for condition 3. ***P*<0.01, **P*<0.05 versus control, by two-way ANOVA followed by Bonferroni's post hoc test. Two-tailed Student's t-test was used in panel (F). n.s. = non-significant.

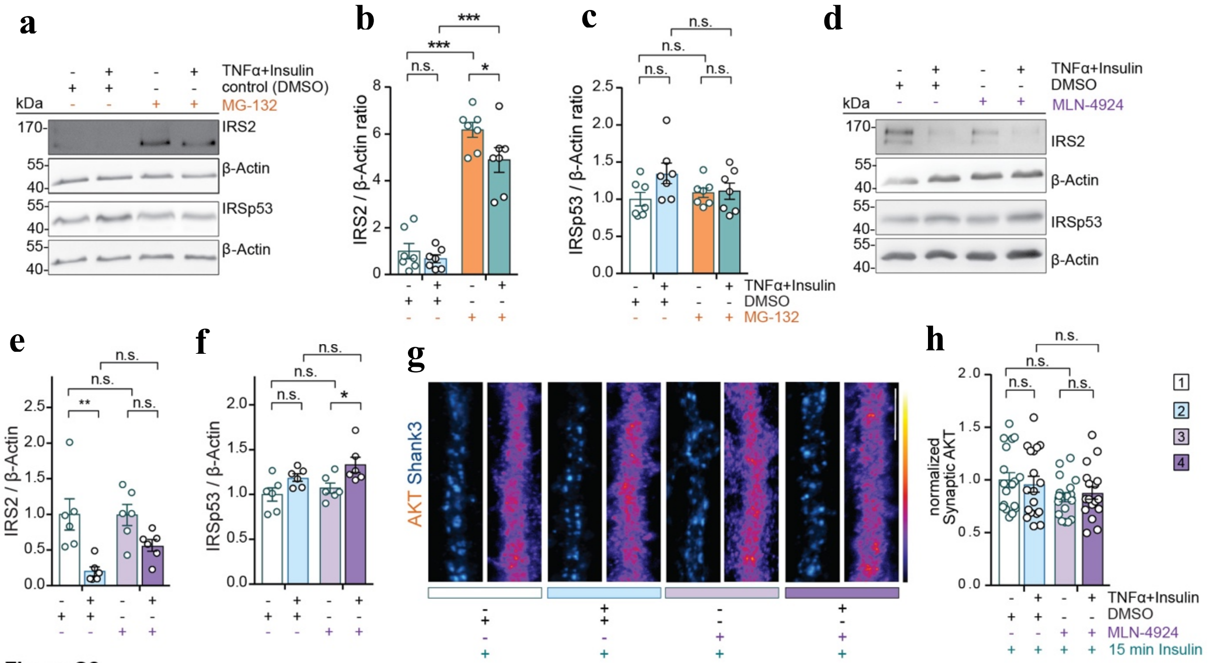

Figure S2

Figure S2. Proteasomal degradation of IRS2 is not affected by neddylation

a-f). Representative immunoblots of total cell extracts from cortical neurons and the corresponding quantification. MG-132 (**a-c**) or MLN-4924 (**d-f**) were co-applied with insulin and TNFα for 24 h. Membranes were probed with antibodies directed against IRS2 or IRSp53. All blots were probed with β-Actin antibody for normalization. Quantification of the total IRS2 (**b**, *n*=7) reveals a significant increase of the total protein level upon MG-132 treatment without alterations in IRSp53 (**c**, *n*=7) protein levels normalized to β-Actin. **e**) Mln-4924 has no effect on the levels of IRS2 (*n*=6) and **e**) elevated levels of IRSp53 (*n*=6). **g-h**). MLN-4924 treatment had no effect on total AKT levels at synapses. Representative images of dendritic fragments of primary hippocampal neurons immunostained with a pan-AKT and Shank3 antibody (**g**). Original pixel intensities from 0 to 255 are represented as a gradient lookup table. **h**) Scatter dot plot representing no differences in pan AKT immunofluorescence in the Shank3 mask. Number of analyzed dendritic segments are depicted. *n*=18 for conditions 1 & 3; *n*= 16 for condition 2; *n*=17 for condition 4. Scale bar is 10 µm. ***P<0.001, **P<0.01, *P<0.05 versus control, by two-way ANOVA followed by Bonferroni's post hoc test. n.s.= non-significant.

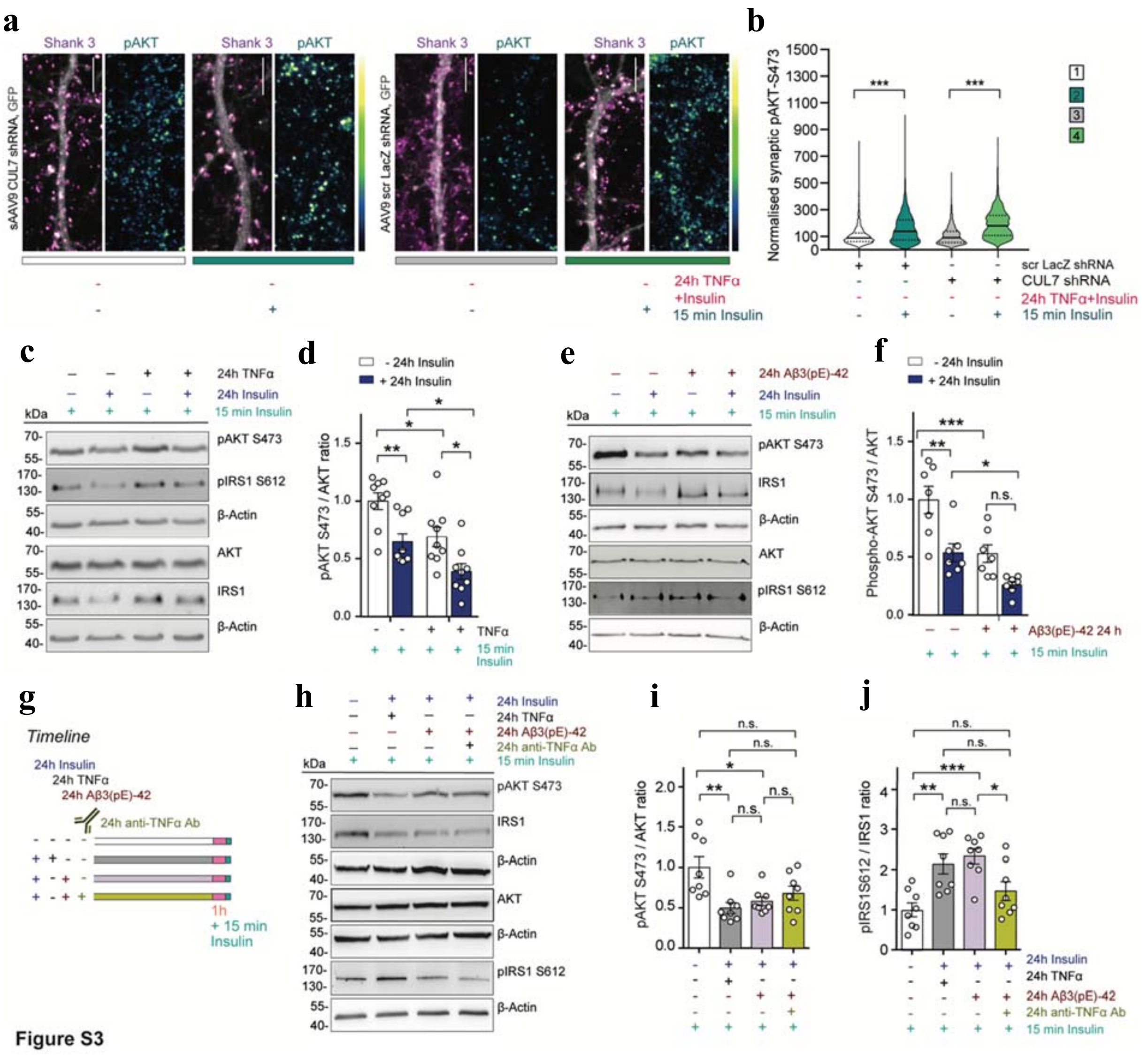

Figure S3. CUL7 KD does not alter insulin signaling and Aβ3(pE) application exacerbates IR

a-b). Stimulation of primary hippocampal neurons infected with AAV9-shRNA CUL7 and AAV9-scr shRNA with insulin for 15 minutes results in activation of AKT under control conditions without application of TNFα/Insulin for 24h. Representative confocal images of dendrites immunolabeled with antibodies directed against pAKT and Shank3. Scale bar is 5 µm. **b**). Violin plot representing means of pAKT immunofluorescence at synaptic sites. ***$P<0.001$ versus control, by two-way ANOVA followed by Bonferroni's post hoc. Data are presented as the mean ± S.E.M. N = number of spines detected by a Shank 3 mask. n= 1353 for condition 1; n= 1602 for condition 2; n= 994 for condition 3; n=1445 for condition 4. **c**). Representative immunoblots of total cortical cell extracts were processed with antibodies against pAKT, AKT, pIRS1 and IRS1. β-Actin was used as a loading control. **d**).

Quantitation of pAKT normalized to AKT levels (*n*=9). **e-f**). Mixed neuronal-glial cultures were treated for 24 h with insulin (100 nM) or Aβ3(pE)-42 (500 nM) or both. After washing for 1 h with medium, cells were stimulated with insulin for 15 min. Western blot analysis and quantitation of total cortical cell extracts probed with antibodies directed against AKT, pAKT, IRS1, and pIRS1. β-Actin was used as a loading control for normalization. **f**). Quantitation of pAKT normalized to AKT levels (*n*=7), $^{***}P<0.001$, $^{**}P<0.01$, $^{*}P<0.05$ versus control, by two-way ANOVA followed by Bonferroni's post hoc test. **g**). Schematic of the experimental protocol where antibodies against TNFα were applied directly into the culture media for 24 hours. **h**). Representative immunoblots of total cortical cell extracts probed with antibodies against pAKT, AKT, pIRS1 and IRS1. Each blot was probed with a β-Actin antibody for normalization. **i-j**). Scatter dot plots show the quantitation of the pAKT/AKT (*n*=8), and pIRS1/IRS1 ratios (*n*=8). $^{***}P<0.001$, $^{**}P<0.01$, $^{*}P<0.05$ versus control, by two-way ANOVA followed by Tukey's multiple comparison test. n.s. = non-significant. Data are presented as the mean ± S.E.M.

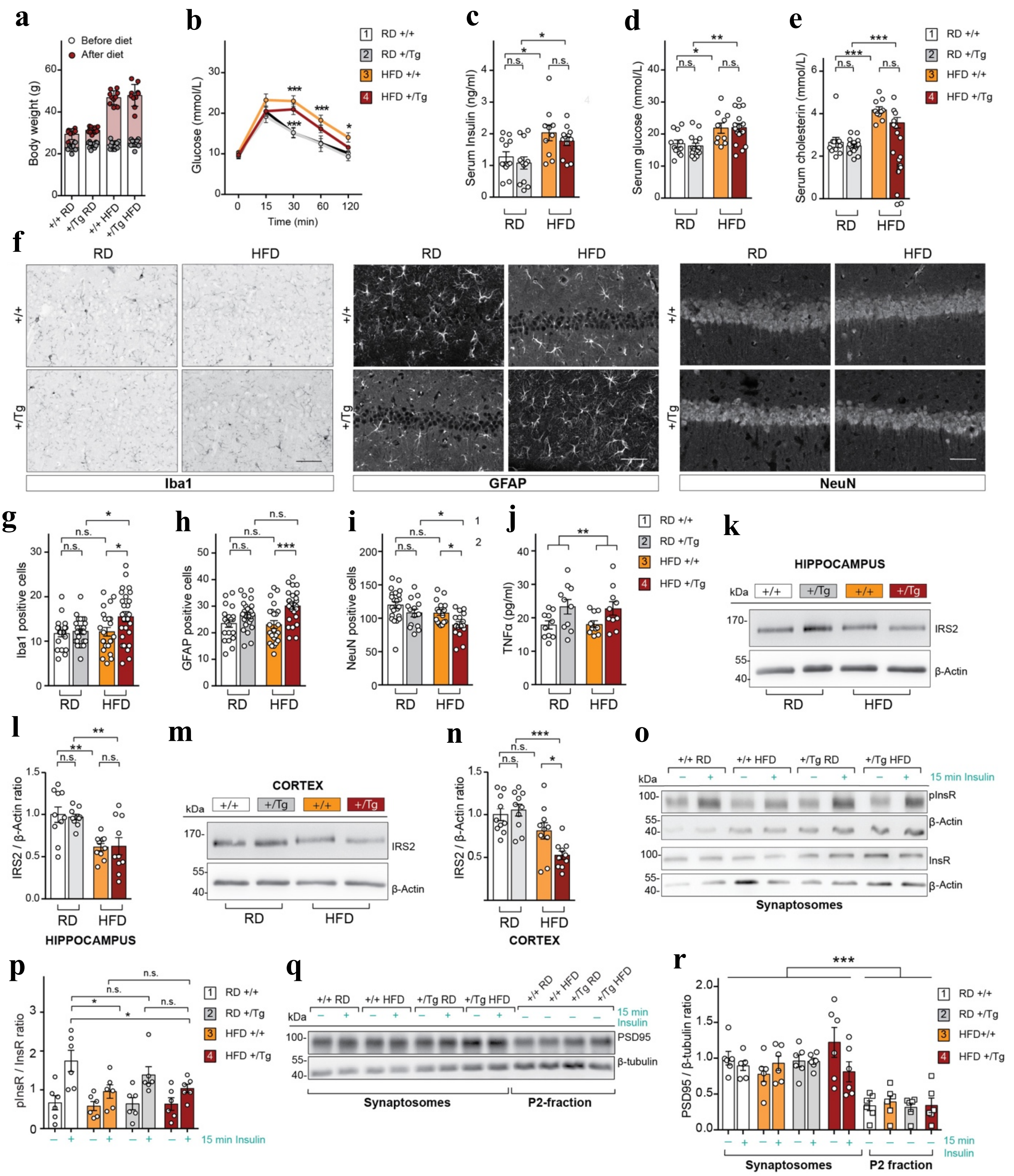

Figure S4

Figure S4. MetS induces neuronal cell loss, astro- and microglia activation in the hippocampal CA1 region of heterozygous TBA2.1 mice

a). Scatter dot plot of the body weight of mice fed with a RD or HFD determined at the beginning of the diet and before starting every experiment; n=8 for condition 1; n=10 for condition 2; n=12 for condition 3; n=10 for condition 4. +/+: wild-type; Tg: Transgene **b**). Time required for clearance of the blood sugar of mice fed with a RD or HFD. **c-e**). No differences were detected in serum insulin (**c**) for conditions 1,2 & 4 n=12; n=10 for

condition 3, glucose (**d**) and cholesterol (**e**) levels between wt and heterozygous TBA2.1 mice fed with a HFD. In both **d** and **e,** *n*=11 for condition 1; *n*=15 for condition 2; *n*=10 for condition 3; *n*=17 for condition 4. **f-j**). Representative images of coronals sections stained for Iba1, GFAP and NeuN (**f**). Scale bar is 100 μm. **g-i**). Quantitation of Iba1(**g**), GFAP(**h**) In both **g** and **h.** N = 20 for condition 1; *n*=26 for conditions 2 & 4; *n*=23 for condition 3. Quantification of NeuN positive cells (**i**) *n*=22 for condition 1; *n*=14 for condition 2; *n*=16 for condition 3; *n*=17 for condition 4. **j**). Irrespective of the diet, TNFα levels in the cortex of heterozygous TBA2.1 mice is significantly higher compared to wt mice as determined from total lysates with ELISA (*n*=10). **k-n**). HFD results in decreased levels of IRS2 in the hippocampus and cortex of heterozygous TBA2.1 mice. IRS2 protein levels were assessed by immunoblots from the total hippocampal or cortical homogenates. β-Actin was used as loading control. **l** (*n*=9); **n** (*n*=10). ****P*<0.001, ***P*<0.01, **P*<0.05 versus control, by two-way ANOVA followed by Bonferroni's post hoc test. **o, p**). Immunoblot analysis of stimulated with insulin synaptosomes isolated from cortices of wt and heterozygous TBA2.1 mice fed with HFD. Membranes were probed with antibodies detecting pInsR (Tyr1150), InsR, and PSD95. Scatter dot plot shows the quantitation of pInsR/InsR ratio (*n*=6). **P*<0.05 versus control, by two-way ANOVA followed by Bonferroni's post hoc test. **q, r**). Representative immunoblots and quantitation of PSD95/β-tubulin ratios indicating similarities in synaptosome preparation between different genotypes fed with a RD and HFD. Synaptosomes isolated from cortices underwent *in vitro* insulin stimulation for 15 min. Stimulated and non-stimulated synaptosomes preparations were loaded onto SDS-PAGE as well as the crude membrane fraction (P2-fraction). (*n*=6). ****P*<0.001, versus control, by unpaired t-Test. n.s. = non-significant. Data are presented as mean ± S.E.M.

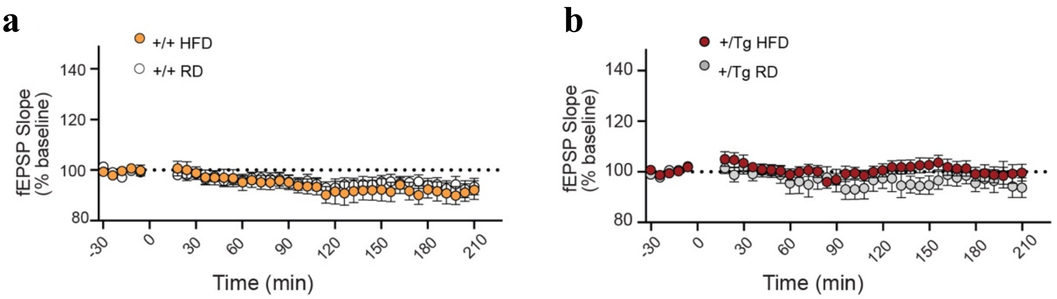

Figure S5

Figure S5. Baseline recordings of fEPSP in hippocampal acute slices from wt and heterozygous TBA2.1 mice fed with a RD or HFD

a, b) The diet and the genotype did not affect the baseline fEPSP slope recorded by the second input on the same slice in which LTD was induced by the first input (see Fig. 5).

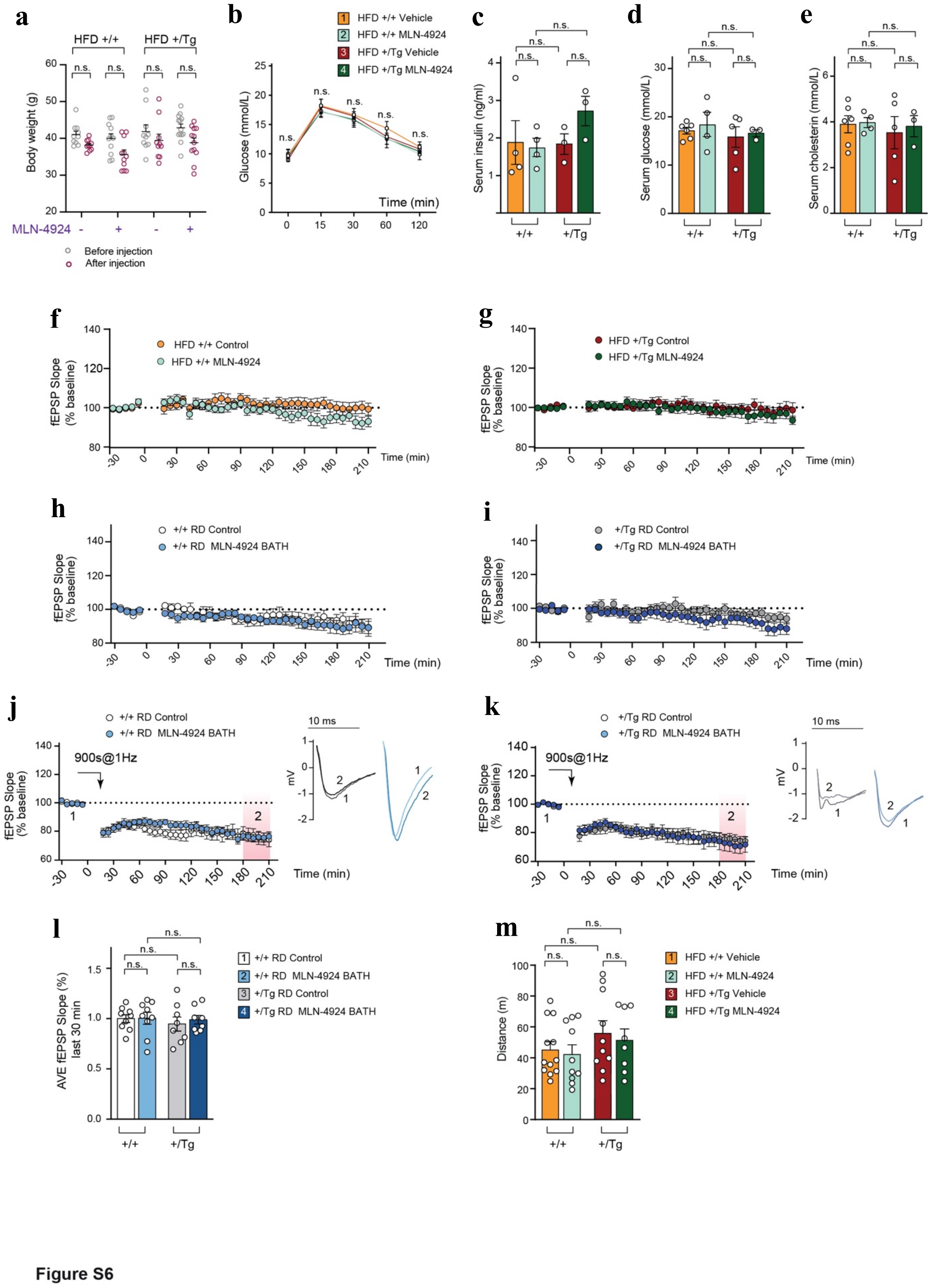

Figure S6. Characterization of the effects of MLN-4924 on basic metabolic parameters, basal fEPSP slope and LTD-dependent learning and memory in WT and heterozygous TBA2.1 mice fed with a RD or HFD

a). Body weight of heterozygous TBA2.1 fed with a HFD and treated either with vehicle or MLN-4924 was measured before and after the injection period of 14 days and the body weight mass is plotted in the scattered dot plot. n=10 for groups 1 & 3; n=11 for group 2; n=13 for group 4. **b**). A glucose tolerance test was performed after 6 h of fasting one day after the last injection with vehicle/MLN-4924. n=8 for group 1; n=9 for groups 2, 3 & 4. Quantitation of insulin (**c**) n=4 for groups 1 & 2; n=4 for group 3 and n=4 for group 4. Quantification of cholesterol (**d**), and glucose (**e**); in both **d** and **e** n=6 for group 2; n=4 for group 3; n=5 for group 3; and n=3 for group 4. Serum levels showed no significant differences between genotypes or drug treatment. n.s. = non-significant versus control, by two-way ANOVA of repeated measures followed by Bonferroni's post hoc test. **f, g**). MLN-4924 injection did not affect baseline fEPSP slope during LTD recording in wt and heterozygous TBA2.1 mice. **h**). Bath application of MLN-4924 did not affect baseline recordings of fEPSP slope during LTD recording in wt or heterozygous TBA2.1 animals fed with a RD. **j, k**). Bath application of MLN-4924 in hippocampal slices from wt or heterozygous TBA2.1 mice fed with a RD did not elicit alterations in plasticity. **l**). Averaged fEPSP slope values measured 180 -210 min after LTD induction showing no differences among the groups; n=8 for groups 1 & 2; n=8 for groups 3 & 4. **m**). Scatter dot plot shows no differences in the total distance walked during the habituation phase; n=12 for group 1; n=10 for group 2; n=10 for group 3; n=8 for group 4. n.s.=non-significant versus control, by two-way ANOVA followed by Bonferroni's post hoc test. Data are presented as the mean ± S.E.M.

Supplementary Table S1. Expression constructs and antibodies used in this study.

RESOURCE	SOURCE	IDENTIFIER	Application
DNA plasmids / constructs			
pCMV-IRS1-GFP	This paper,<br>subcloned from pBlu2KSP-IRS1	RRID: Addgene_11027	Co-IP
pCMV-IRS2-GFP	This paper,<br>subcloned from pBABE-Puro-IRS2-Myc	RRID: Addgene_11373	Co-IP
pAAV-CMV-GFP-P2A-CBD-6xHIS-IRS1	This paper	N/A	PD
pcDNA3-myc3-CUL1	Ohta et. al., Mol Cell.1999, 3(4):535-41	RRID: Addgene_19896	Co-IP
pcDNA3-myc3-CUL2	Ohta et. al., Mol Cell.1999, 3(4):535-41	RRID: Addgene_19892	Co-IP
pcDNA3-myc-CUL3	Ohta et. al., Mol Cell.1999, 3(4):535-41	RRID: Addgene_19893	Co-IP
pcDNA3-MYC3-CUL4A	Ohta et. al., Mol Cell.1999, 3(4):535-41	RRID: Addgene_19951	Co-IP
pcDNA3-MYC3-CUL4B	Hu et. al., Genes Dev.2008, 22(7):886-871	RRID: Addgene_19922	Co-IP
pcDNA3-myc-CUL 5	Ohta et. al., Mol Cell.1999, 3(4):535-41	RRID: Addgene_19895	Co-IP
pcDNA3-myc-CUL 7	Andrews et al., Oncogene. 2006 25(33): 4534-4548	RRID: Addgene_20695	Co-IP
pAAV-CMV-HA-NEDD8	This paper,<br>subcloned from<br>HA-NEDD8 (Kamitani et. al., JBC, 1997. 272(45): 28557-6) into pAAV-CMV	RRID: Addgene_18711<br>Agilent #240071	Co-IP
pAAV-pCMV-HA	This paper	RRID: N/A	Co-IP
CUL7 shRNA<br>targeting sequence:<br>5`GATGAGATCTATGCC AACTG 3`	This paper	RRID: N/A<br><br>Subcloned into<br>RRID: Addgene_92155	ICC
scr LacZ shRNA<br>targeting sequence:<br>5`AATTTAACCGCCAGT CAGGCT 3`	This paper	RRID: N/A<br><br>Subcloned into<br>RRID: Addgene_92155	ICC

Primary Antibodies			
anti-(pan) Akt (40D4) monoclonal mouse Ab	Cell Signaling Technology	Cat.: #2920<br>RRID: AB_11476220	ICC 1:500<br>WB 1:2000
anti-phospho AKT/Ser473 polyclonal rabbit Ab	Cell Signaling Technology	Cat.: #9271S<br>RRID: AB_329826	ICC1:150<br>WB 1:2000
anti- human Amyloid beta (N) (82E1) monoclonal mouse Ab	IBL International	Cat.: #10323<br>RRID: AB_1630806	IHC 1:200
anti-Aβ-pE3-218003 polyclonal rabbit Ab	SySy	Cat.: #218003<br>RRID: AB_2056424	IHC 1:200
anti-CUL7 (Ab38)	Sigma-Aldrich	Cat.: #C1743	WB 1:1000

monoclonal mouse Ab		RRID: AB_796201	
anti-NEDD8<br>polyclonal rabbit Ab	Proteintech	Cat.: #16777-1-AP<br>RRID: AB_10598467	WB 1:2000
anti-NeuN (A60)<br>monoclonal mouse Ab	Merk Millipore	Cat.: #MAB377<br>RRID: AB_2298772	ICC 1:500
anti-PI3 Kinase p110γ<br>polyclonal rabbit Ab	Cell Signaling Technology	Cat.: #4252<br>RRID: AB_329871	WB 1:1000
anti-PI3Kinase p110γ (D55D5)<br>monoclonal rabbit Ab	Cell Signaling Technology	Cat.: #5405<br>RRID: AB_1904087	endogenous IP 1:200
anti-Iba1<br>polyclonal guinea pig Ab	SySy	Cat.: #234004<br>RRID: AB_2493179	ICC 1:300
anti-Homer1<br>monoclonal mouse Ab	SySy	Cat.: #160011<br>RRID: N/A	ICC 1:500
anti-phospho-IGF-1 Receptor β (Tyr1135/1136)/Insulin Receptor β (Tyr1150-1151) (19h7) monoclonal rabbit Ab	Cell Signaling Technology	Cat.: #3024<br>RRID: AB_331253	WB 1:1000
anti-Insulin Receptor β (4B8) monoclonal rabbit Ab	Cell Signaling Technology	Cat.: #3025<br>RRID: AB_2280448	WB 1:1000
anti-IRS1 Rb	Merk Millipore	Cat.: #06-248<br>RRID: AB_2127890	WB 1:500
anti-IRS2 (9.5.2)<br>monoclonal mouse Ab	Merk Millipore	Cat.: #MABS15<br>RRID: AB_10615782	WB 1:1000
anti-phospho-IRS1 /Ser 612 Rabbit monoclonal Ab	Cell Signaling Technology	Cat.: #3203<br>RRID: AB_1031167	WB 1:1000
anti-IRSp53<br>polyclonal rabbit Ab	Merk Millipore	Cat.: #07-786<br>RRID: AB_612039	WB 1:1000
anti-phospho-PKR /Thr451<br>polyclonal rabbit Ab	Merk Millipore	Cat.: #07-886<br>RRID: AB_568879	WB 1:1000
anti-TNF-alpha<br>polyclonal goat Ab	R&D system	Cat.: #AF-510-NA<br>RRID: AB_354511	TNF-alpha neutralization 1:500
anti-mono- and polyubiquitinylated conjugates (FK2)<br>monoclonal mouse Ab	Enzo Life Science	Cat.: #BML-PW8810<br>RRID: AB_10541840	WB 1:1000
anti-Shank3<br>polyclonalrabbit Ab	SySy	Cat.: #162302<br>RRID: AB2619862	WB 1:1000<br>ICC 1:500
anti-Shank3<br>polyclonal guinea pig Ab	SySy	Cat.: #162304<br>RRID: AB2619863	ICC 1:500
anti-PSD95(K28/43)<br>monoclonal mouse Ab	Neuromab	Cat.: #75-028<br>RRID: AB_2292909	WB 1:1000 ICC 1:500
anti-beta-actin (AC-15)<br>monoclonal mouse Ab	Sigma	Cat.: #A-5441<br>RRID: 476744	WB 1:3000
anti-GFAP<br>polyclonal rabbit Ab	Sigma-Aldrich	Cat.: #G-9269<br>RRID: AB_477035	IHC 1:400
anti-MAP2 (AP20)<br>monoclonal mouse Ab	Millipore	Cat.: #MAB3418<br>RRID: AB_94856	ICC 1:500
anti-MAP2-AlexaFluor 488 conjugated monoclonal	Millipore	Cat.: #MAB3418x<br>RRID: AB11212966	ICC 1:500

mouse Ab			
anti-Myc tag (9B11) monoclonal mouse Ab	NEB/Cell Signaling Technology	Cat.: #2276 S RRID: AB_94856	WB 1:1000
anti-HA monoclonal rat Ab	Roche	Cat.: #11867423001 Cat#11867423001; RRID: AB_390918	WB 1:1000
anti-GFP Epitope Tag (B34) monoclonal mouse Ab	BioLegend	Cat.: #902601 RRID: AB_2565021	Heterologous Co-IP, WB 1:1000
Secondary Antibodies			
Goat anti-rabbit-AlexaFluor 488	Thermo Fisher Scientific	Cat.: #A-11034 RRID: AB_25766217	ICC/IHC 1:500
Donkey anti-rabbit-AlexaFluor 488	Molecular Probes	Cat.: #A-21206 RRID: AB_2535792	ICC/IHC 1:500
Goat anti-rabbit-AlexaFluor 568	Thermo Fisher Scientific	Cat.: #A-11036 RRID: AB_10563566	ICC/IHC 1:500
Goat-anti-rabbit-Cy5	Jackson Immuno Research	Cat.: #111-175-144 RRID: AB_2338013	ICC/IHC 1:500
Goat-anti-rabbit-AlexaFluor 633	Molecular Probes	Cat.: #A-21070 RRID: AB_2535731	ICC/IHC 1:500
Goat anti-mouse-AlexaFluor 488	ThermoFisher Scientific	Cat.: #A-11001 RRID: AB_2534069	ICC/IHC 1:500
Goat anti-mouse-AlexaFluor 568	ThermoFisher Scientific	Cat.: #A-11031 RRID: AB_144696	ICC/IHC 1:500
Goat anti-mouse-AlexaFluor 647	Thermo Fisher Scientific	Cat.: #A-21236 RRID: AB_2535805	ICC/IHC 1:500
Donkey anti-mouse-AlexaFluor 647	Molecular probes	Cat.: #A-31571 RRID: AB_162542	ICC/IHC 1:500
Goat Anti-guinea pig-AlexaFluor405	Abcam	Cat.: #Ab175678 RRID: 282755	ICC/IHC 1:500
Goat anti-guinea pig-AlexaFluor 488	Molecular Probes	Cat.: #A-11073 RRID: AB_2534117	ICC 1:500
Goat anti-guinea pig-AlexaFluor 568	Invitrogen	Cat.: #A-11075 RRID: AB_141954	ICC 1:500
Goat Anti-guinea pig-AlexaFluor 647	Thermo Fisher Scientific	Cat.: #A-21450 RRID: AB_141882	ICC/IHC 1:500
Goat Anti-Rat IgG Ab, HRP conjugate	Sigma Aldrich	Cat.: #AP136P RRID: AB_11214444	WB 1:5000
Goat Anti Mouse IgG Ab	Jackson Immuno Research	Cat.: #115-035-146 RRID: AB_2307392_	WB 1:20000
Goat Anti rabbit igG Ab	Jackson Immuno Research	Cat.: #115-035-144 RRID: AB_2307391	WB 1:20000
